# Human inherited PD-L1 deficiency is clinically and immunologically less severe than PD-1 deficiency

**DOI:** 10.1084/jem.20231704

**Published:** 2024-04-18

**Authors:** Matthew B. Johnson, Masato Ogishi, Clara Domingo-Vila, Elisa De Franco, Matthew N. Wakeling, Zineb Imane, Brittany Resnick, Evangelia Williams, Rui Pedro Galão, Richard Caswell, James Russ-Silsby, Yoann Seeleuthner, Darawan Rinchai, Iris Fagniez, Basilin Benson, Matthew J. Dufort, Cate Speake, Megan E. Smithmyer, Michelle Hudson, Rebecca Dobbs, EXE-T1D consortium, Michael N. Weedon, Bart O. Roep, William Hagopian, Zoe Quandt, Andrew T. Hattersley, Peng Zhang, Stephanie Boisson-Dupuis, Mark S. Anderson, Jean-Laurent Casanova, Timothy I. Tree, Richard A. Oram

**Affiliations:** 1 Clinical and Biomedical Sciences, Faculty of Health and Life Sciences, University of Exeter, Exeter, UK; 2 St. Giles Laboratory of Human Genetics of Infectious Diseases, Rockefeller Branch, The Rockefeller University, New York, NY, USA; 3 Department of Immunobiology, School of Immunology and Microbial Sciences, Kings College London, London, UK; 4 Faculty of Medicine and Pharmacy, Mohammed 5 University of Rabat, Rabat, Morocco; 5 National Institute for Health and Care Research Exeter Clinical Research Facility, Royal Devon University Healthcare NHS Foundation Trust, Exeter, UK; 6 Department of Infectious Diseases, School of Immunobiology and Microbial Sciences, Kings College London, London, UK; 7 Laboratory of Human Genetics of Infectious Diseases, Necker Branch, INSERM U1163, Paris, France; 8 Imagine Institute, Paris Cité University, Paris, France; 9 Center for Systems Immunology, Benaroya Research Institute, Seattle, WA, USA; 10 Center for Interventional Immunology, Benaroya Research Institute, Seattle, WA, USA; 11 Endocrine Division, Department of Medicine, University of California San Francisco, San Francisco, CA, USA; 12 Diabetes Center, University of California San Francisco, San Francisco, CA, USA; 13 Department of Pediatrics, Necker Hospital for Sick Children, Paris, France; 14 Howard Hughes Medical Institute, New York, NY, USA

## Abstract

We previously reported two siblings with inherited PD-1 deficiency who died from autoimmune pneumonitis at 3 and 11 years of age after developing other autoimmune manifestations, including type 1 diabetes (T1D). We report here two siblings, aged 10 and 11 years, with neonatal-onset T1D (diagnosed at the ages of 1 day and 7 wk), who are homozygous for a splice-site variant of *CD274* (encoding PD-L1). This variant results in the exclusive expression of an alternative, loss-of-function PD-L1 protein isoform in overexpression experiments and in the patients’ primary leukocytes. Surprisingly, cytometric immunophenotyping and single-cell RNA sequencing analysis on blood leukocytes showed largely normal development and transcriptional profiles across lymphoid and myeloid subsets in the PD-L1-deficient siblings, contrasting with the extensive dysregulation of both lymphoid and myeloid leukocyte compartments in PD-1 deficiency. Our findings suggest that PD-1 and PD-L1 are essential for preventing early-onset T1D but that, unlike PD-1 deficiency, PD-L1 deficiency does not lead to fatal autoimmunity with extensive leukocytic dysregulation.

## Teaser

Johnson, Ogishi, and Domingo-Vila et al. describe two siblings with inherited PD-L1 deficiency. Human PD-L1 deficiency underlies early-onset T1D, like PD-1 deficiency, but does not lead to fatal autoimmunity with extensive leukocytic dysregulation, unlike PD-1 deficiency.

## Graphical Abstract

**Figure:**
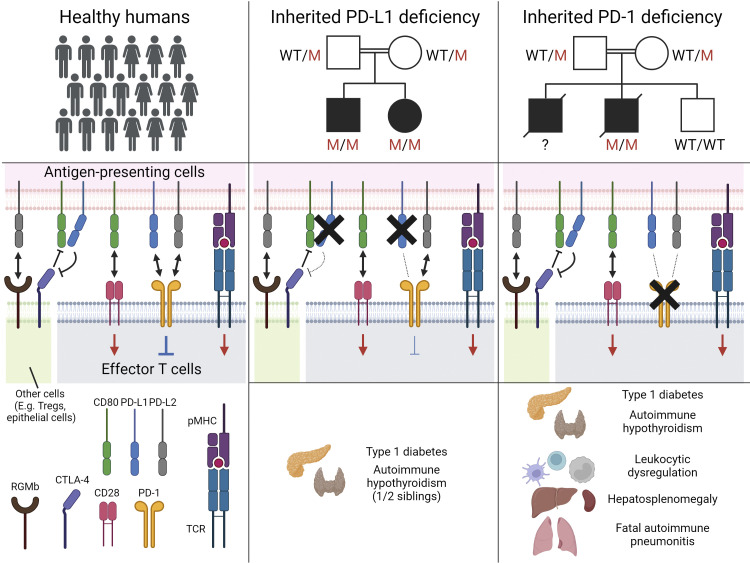

## Introduction

Autoimmune diseases are a growing public health problem estimated to affect ∼10% of the population in the United Kingdom (Conrad et al., 2023). Monogenic etiologies of autoimmunity have provided unique insights into the physiological mechanisms governing self-tolerance in humans (Bousfiha et al., 2022; Notarangelo et al., 2020). We recently described two siblings with autosomal recessive (AR) complete programmed death 1 (PD-1) deficiency, both of whom had type 1 diabetes (T1D) and thyroiditis in childhood and died from autoimmune pneumonitis at the ages of 3 and 11 years (Ogishi et al., 2021). The clinical and cellular phenotypes of the PD-1-deficient proband—including T1D, thyroiditis, pneumonitis, hepatosplenomegaly, and high levels of CD4^−^CD8^−^ double-negative αβ T cells—resembled those of patients heterozygous for *STAT3* GOF variants (Fabre et al., 2019). PD-1 is a coinhibitory checkpoint expressed most strongly on activated T and B lymphocytes, followed by resting T/B/natural killer (NK) lymphocytes and myeloid cells (Sharpe and Pauken, 2018). Neutralizing antibodies against human PD-1 have been successfully used in immunotherapy for multiple cancers and have substantially improved therapeutic outcomes (Chamoto et al., 2023). Nevertheless, a significant minority of patients on PD-1 blockade develop immune-related adverse events (irAEs)—i.e., autoimmunity—including neurological, intestinal, pulmonary, hepatic and renal disease, and endocrinopathy (Martins et al., 2019). Despite the widespread use of PD-1 blockade in cancer immunotherapy, the systemic impact of PD-1 blockade on the development and function of diverse leukocyte subsets other than intratumoral antigen-specific T lymphocytes remains largely unknown. We previously reported high counts of CD4^−^CD8^−^ double-negative αβ T cells with high levels of Ki67 (a proliferation marker) expression in two cohorts of cancer patients 1 mo after PD-1 blockade monotherapy (Ogishi et al., 2021). This phenotype is consistent with the expansion and activation of this subset in both the PD-1-deficient child and the PD-1 knockout (KO) mice studied, hinting at a possible mechanism of leukocyte dysregulation in the absence of PD-1 common to mice and humans.

PD-1 exerts a coinhibitory signal that suppresses the activation of lymphocytes engaged with two known ligands, programmed death ligands 1 and 2 (PD-L1 and PD-L2, encoded by *CD274* and *PDCD1LG2*, respectively) (Sharpe and Pauken, 2018). PD-L1 is known to be expressed not only on activated T and B lymphocytes, monocytes, macrophages, and dendritic cells (DCs) but also on epithelial cells, vascular endothelial cells, and pancreatic β cells, particularly under inflammatory conditions (Sharpe and Pauken, 2018). By contrast, PD-L2 is known to be expressed only on certain types of DCs, macrophages, and B lymphocytes (Sharpe and Pauken, 2018). Despite its restricted expression, PD-L2 interacts with PD-1 with an affinity four times greater than that of PD-L1 (Zak et al., 2017). PD-L1 and PD-L2 can also bind CD80 and the repulsive guidance molecule b (RGMb), respectively (Sharpe and Pauken, 2018). PD-L1 expressed on antigen-presenting cells interacts with CD80 in *cis*, which restricts the inhibitory PD-1:PD-L1 signals while protecting CD80 from cytotoxic T lymphocyte-associated antigen 4 (CTLA-4)–mediated trans-endocytosis, thereby strengthening CD80:CD28 costimulatory signals (Sugiura et al., 2019; Zhao et al., 2019). Conversely, PD-L2:RGMb interaction is thought to be costimulatory for the CD4^+^ T-helper 1 response and, thus, to inhibit CD4^+^ T-helper 2–mediated airway inflammation (Nie et al., 2018; Xiao et al., 2014). It is also possible that RGMb serves as a decoy receptor for PD-L2 to limit the inhibitory PD-1:PD-L2 signals in a physiological context.

PD-L1 blockade by neutralizing antibodies has also been used in cancer immunotherapy (Chamoto et al., 2023). The clinical benefits of PD-L1 blockade are thought to result from the unleashing of cytotoxic T lymphocytes from inhibition by the PD-L1 expressed on DCs (Oh et al., 2020; Peng et al., 2020). Conversely, attempts at PD-L2 blockade in humans have been unsuccessful to date (Chamoto et al., 2023). PD-L1 blockade triggers irAEs similar to those observed during PD-1 blockade, including T1D (Bluestone et al., 2018; Kotwal et al., 2019). Importantly, the blockade of either PD-1 or PD-L1 can trigger rapid-onset T1D, whereas antibody-mediated neutralization of CTLA-4 does not seem to cause T1D (Quandt et al., 2020), strongly suggesting a specific and indispensable role of PD-1:PD-L1 signaling in pancreatic islet homeostasis. Moreover, NOD mouse models lacking PD-L1 or PD-L2 and bone marrow chimera experiments have provided additional evidence that the PD-L1 expressed on non-hematopoietic cells is essential for preventing rapid-onset T1D in mice, whereas PD-L2 is not (Keir et al., 2006). However, it remains unclear whether both these ligands are indispensable or mutually redundant for preventing various forms of autoimmunity in humans. We hypothesized that the identification and characterization of patients with inherited PD-L1 or PD-L2 deficiency would help to address this question and provide insight into the mechanisms of autoimmunity triggered by blockades of PD-1 or PD-L1.

## Results

### Two siblings with neonatal-onset type 1 diabetes

**Figure S1.**
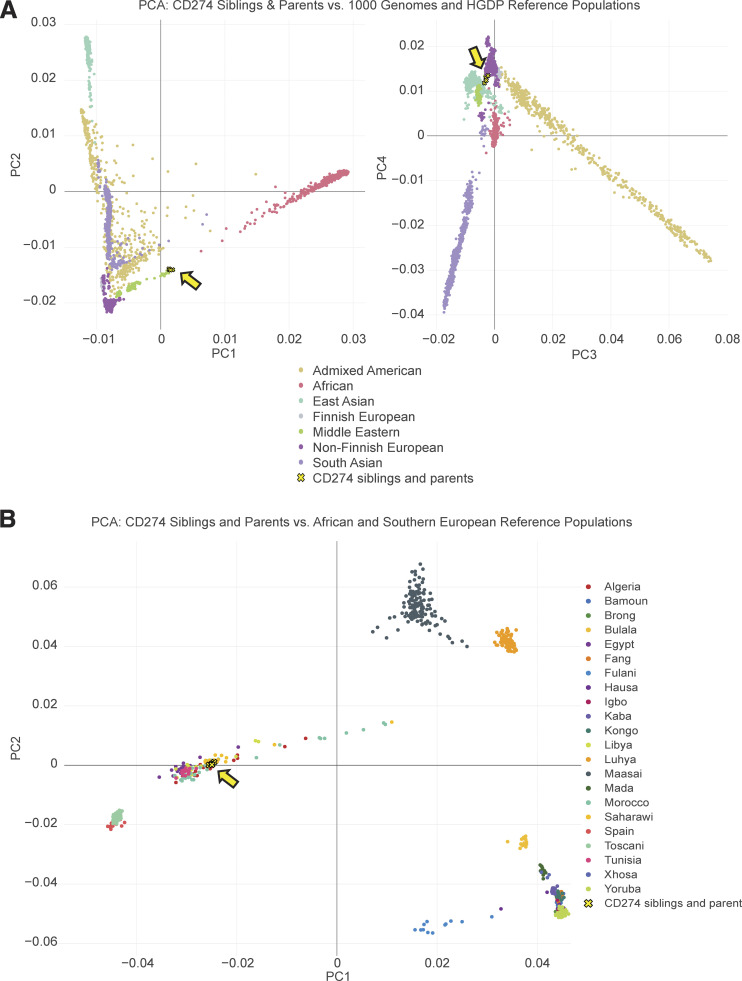
**Genetic analysis of the two siblings with neonatal-onset type 1 diabetes. (A)** Ancestry-level PCA. PC scores were computed from the Human Genome Diversity Project and 1,000 Genomes phase 3 global genomic reference population datasets (Auton et al., 2015; Bergström et al., 2020) and local WGS data for the two affected siblings and their healthy relatives. The PCA plot is colored according to the reported genetic ancestry group (for controls). Individuals from the family studied are represented as yellow crosses outlined in black. **(B)** Country-level analysis. The previously reported dataset (Henn et al., 2012) was used as a reference. The PCA plot is colored according to the reported country of origin (for controls). Individuals from the family studied are represented as yellow crosses outlined in black.

We studied two siblings, a male proband and his younger sister (aged 11 and 10 years, respectively, at the time of the study), born to second-degree consanguineous parents of Moroccan descent, as confirmed by principal component analysis (PCA) (Fig. S1, A and B). Both developed neonatal-onset T1D (diagnosed at the ages of 1 day and 7 wk, respectively). The male proband was subsequently diagnosed with asthma at the age of 5 mo, autoimmune hypothyroidism at the age of 3 years, and growth hormone (GH) deficiency at the age of 10 years. He also had mild intellectual disability with delayed language development. By contrast, his sister had no clinical manifestations other than T1D. Both individuals tested negative for anti-islet autoantibodies (antibodies against GAD, IA-2, or ZnT8) at the ages of 11 and 10 years, respectively (samples not available at initial diagnosis). Levels of random C-peptide, excreted at a concentration equimolar to endogenous insulin, were extremely low (5 and 4 pmol/liter, respectively; normal range: >200 pmol/liter), consistent with complete insulin deficiency. The siblings are currently on insulin at a full replacement dose and have no additional complications related to their diabetes. Whole-genome sequencing (WGS) and targeted sequencing ruled out all known genetic causes of neonatal diabetes (De Franco et al., 2015). Both individuals and their mother are heterozygous for the common T1D risk HLA haplotype DR4 (DRB1*04:05-DQB1*03:02) (Noble and Valdes, 2011). The affected girl also carries the T1D-protective DQ6 (DRB1*15:02-DQB1*06:01) haplotype, whereas other alleles carried by the male proband and the mother are neutral with respect to T1D risk. Given the extremely early presentation of diabetes in the two siblings and their consanguinity, we hypothesized that a single biallelic pathogenic variant present in both siblings or a previously unidentified etiological gene was likely to underlie their neonatal-onset T1D.

### A private homozygous splice-site variant of *CD274*

**Figure 1.**
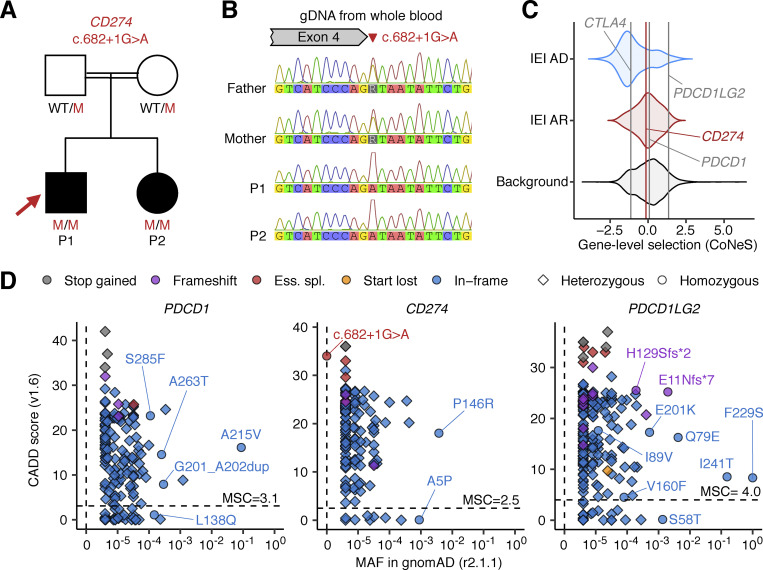
**Two siblings homozygous for a splice-site variant of *CD274*. (A)** The pedigree. Black symbols indicate affected individuals. Genotypes for the *CD274* allele are also shown. WT: wild-type. M: mutant. E?: unknown. **(B)** Validation of the variant by Sanger sequencing. **(C)** Gene-level negative selection. *PDCD1*, *CD274*, and *PDCD1LG2* (encoding PD-1, PD-L1, and PD-L2, respectively) are not under negative selection, as shown by CoNeS score (Rapaport et al., 2021), as also reported for other genes for which mutations underlie AR IEI. *CTLA4* is also shown, as an example of another gene under negative selection. **(D)** Population genetics of *PDCD1*, *CD274*, and *PDCD1LG2*. The MAF and CADD scores for all non-synonymous variants found in the gnomAD database are depicted. All biallelic variants are labeled with their predicted protein-level consequences. The horizontal dotted line indicates the MSC (Itan et al., 2016; Kircher et al., 2014).

WGS revealed that both siblings were homozygous for a private splice-donor (SD) variant in the fourth intron of *CD274* (NM_014143:c.682+1G>A) (Fig. 1 A) (sequencing metrics in Table S1). The G nucleotide present in this position in the wild-type (WT) sequence is strongly conserved (Zhang et al., 2022). The combined annotation-dependent depletion (CADD) score (Kircher et al., 2014) of the variant was 34.0, well above the mutation significance cutoff (MSC) (Itan et al., 2016) for *CD274* of 2.5. Moreover, SpliceAI (Jaganathan et al., 2019) predicted an extremely high probability (score = 0.98) of the variant disrupting the SD site. Sanger sequencing confirmed that the affected siblings were homozygous for the *CD274* variant, whereas their parents were heterozygous carriers, consistent with an AR mode of inheritance (Fig. 1, A and B). The homozygosity rate calculated from WGS data was high, consistent with the reported consanguinity of the patients. The two siblings had four other rare homozygous non-synonymous variants in common (GnomAD MAF < 0.01; three missense variants and one three-amino acid duplication) (Table S2). However, none of these variants affected a gene known to cause an immune-related phenotype. We then analyzed all rare variants common to the proband and his sister within the coding sequences or flanking intronic regions of genes known to underlie IEI in a recessive or dominant manner (Table S3 and Materials and methods); neither patient was homozygous or heterozygous for any such variants of these genes (Table S4). We also ruled out potential compound heterozygous inheritance or germline mosaicism resulting in dominant inheritance by sequencing the parents and analyzing each sibling as part of a parent-offspring trio; no shared compound heterozygous or “de novo” variants were identified (Table S5). The male proband had a de novo duplication at 7q11.23 (Table S6), which has been reported to underlie neurological phenotypes, such as epilepsy and mental retardation, but has never been associated with an (auto)immune phenotype (Mervis et al., 2015). His sister did not have this de novo duplication, potentially accounting for the abnormal neurological development of the male proband and the normal development of his sister. These observations suggest that the homozygous splice-site variant of *CD274* may cause AR PD-L1 deficiency and underlie neonatal-onset T1D in the two affected siblings.

### Population genetics of *PDCD1*, *CD274*, and *PDCD1LG2*

Like other genes with deleterious variants underlying AR inborn errors of immunity (IEI), *CD274*, *PDCD1LG2*, and *PDCD1* are not under strong negative selection according to CoNeS (Rapaport et al., 2021) (Fig. 1 C). We investigated the occurrence of pLOF variants of the human *PDCD1*, *CD274*, and *PDCD1LG2* genes in the heterozygous or homozygous state in the general population. The gnomAD database of 141,456 individuals (https://gnomad.broadinstitute.org) contains no individuals homozygous for pLOF variants of *PDCD1* or *CD274*, and only eight individuals homozygous for pLOF variants of *PDCD1LG2* (Fig. 1 D). This database contains 7, 8, and 21 pLOF variants of *PDCD1*, *CD274*, and *PDCD1LG2*, with cumulative minor allele frequencies (MAFs) of 7.1 × 10^−5^, 6.0 × 10^−5^, and 2.7 × 10^−3^, respectively. The frequency of biallelic pLOF genotypes was therefore estimated at 5.0 × 10^−9^, 3.6 × 10^−9^, and 7.4 × 10^−6^, respectively. Likewise, in the UK Biobank WES dataset for 470,000 individuals (https://www.ukbiobank.ac.uk/enable-your-research/about-our-data/genetic-data), 13, 9, and 30 pLOF variants of *PDCD1*, *CD274*, and *PDCD1LG2* were identified, with cumulative MAFs of 3.5 × 10^−5^, 1.4 × 10^−5^, and 1.4 × 10^−3^, respectively. The frequency of biallelic pLOF genotypes was therefore estimated at 1.2 × 10^−9^, 1.9 × 10^−9^, and 2.0 × 10^−6^, respectively. These observations suggest that, like inherited PD-1 deficiency, inherited PD-L1 deficiency is extremely rare in the general population. This rarity is consistent with our hypothesis that homozygosity for the ultrarare splice-site variant of *CD274* in our siblings may result in AR PD-L1 deficiency and underlie neonatal-onset T1D.

### The patients’ *CD274* allele results in an in-frame deletion due to alternative splicing

**Figure 2.**
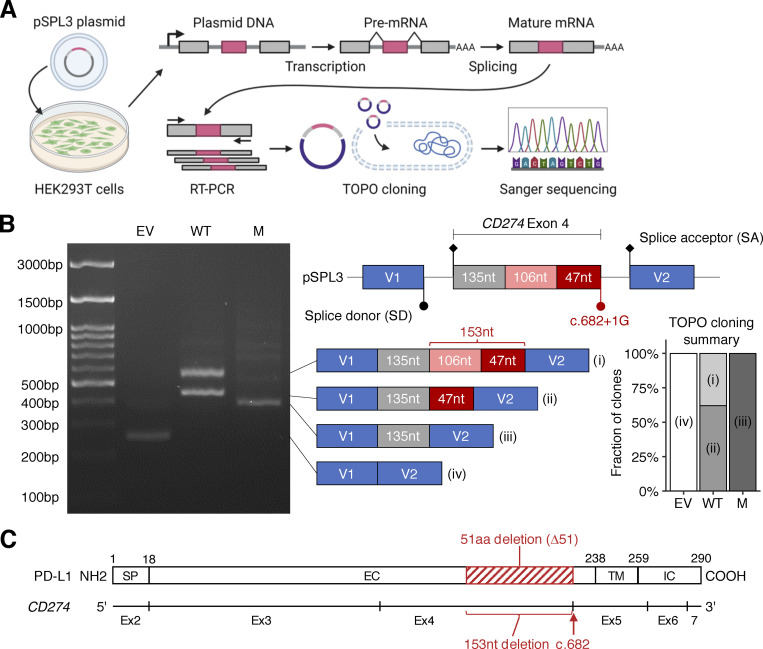
**Analysis of the effect of the *CD274* splice-site variant on mRNA splicing in an overexpression system. (A)** Schematic diagram of an exon-trapping assay. A region of genomic DNA flanking the fourth exon of the canonical *CD274* isoform with or without the c.682+1G>A splice-site in the homozygous state was inserted into the pSPL3 vector. The plasmids were used to transfect HEK293T cells and, 24 h later, the spliced mRNA product was recovered by RT-PCR and TOPO cloning, and subjected to Sanger sequencing. **(B)** Exon trapping. The schematic diagram shows the four types of cDNA identified, with the number of nucleotides in each region indicated. Representative data from two experiments are shown. **(C)** A schematic diagram of the *CD274* mRNA and PD-L1 protein. Exon 1 is omitted because it contains no coding sequence. The red rectangle depicts the 51-amino acid in-frame deletion caused by the c.682+1G>A variant. SP, signal peptide; EC, extracellular domain; TM, transmembrane domain; IC, intracellular domain. Source data are available for this figure: SourceData F2.

We first investigated whether the splice-site variant affected the splicing of *CD274* mRNA. We performed an exon-trapping assay with pSPL3 vectors containing the genomic region flanking the position of the biallelic splice-site variant subcloned from the proband (mutant/mutant) or a pediatric control (WT/WT) (Fig. 2 A). Exon trapping showed a 153-base in-frame deletion (NM_014143:r.530_682del) in all 42 colonies of mutant-transfected HEK293T cells sequenced (Fig. 2, B and C). By contrast, WT-transfected cells produced two transcripts: a canonical transcript (11/29) and an alternative transcript with a deletion of 106 bases (NM_014143:r.530_635del; 18/29) (Fig. 2 B). This 106-base deletion leads to a frameshift predicted to result in premature termination (truncation) due to the presence of a stop codon at position 179. Interestingly, both the r.530_682del and r.530_635del transcripts used the same cryptic SD site. Thus, the patients’ splice-site variant disrupts the canonical SD site essential for the expression of the canonical *CD274* isoform by partially outcompeting the naturally existing cryptic SD site. These data suggest that the patients’ *CD274* allele results in the exclusive expression of an alternative PD-L1 protein isoform carrying an in-frame deletion.

### The alternative PD-L1 protein isoform is weakly expressed in an overexpression system

**Figure 3.**
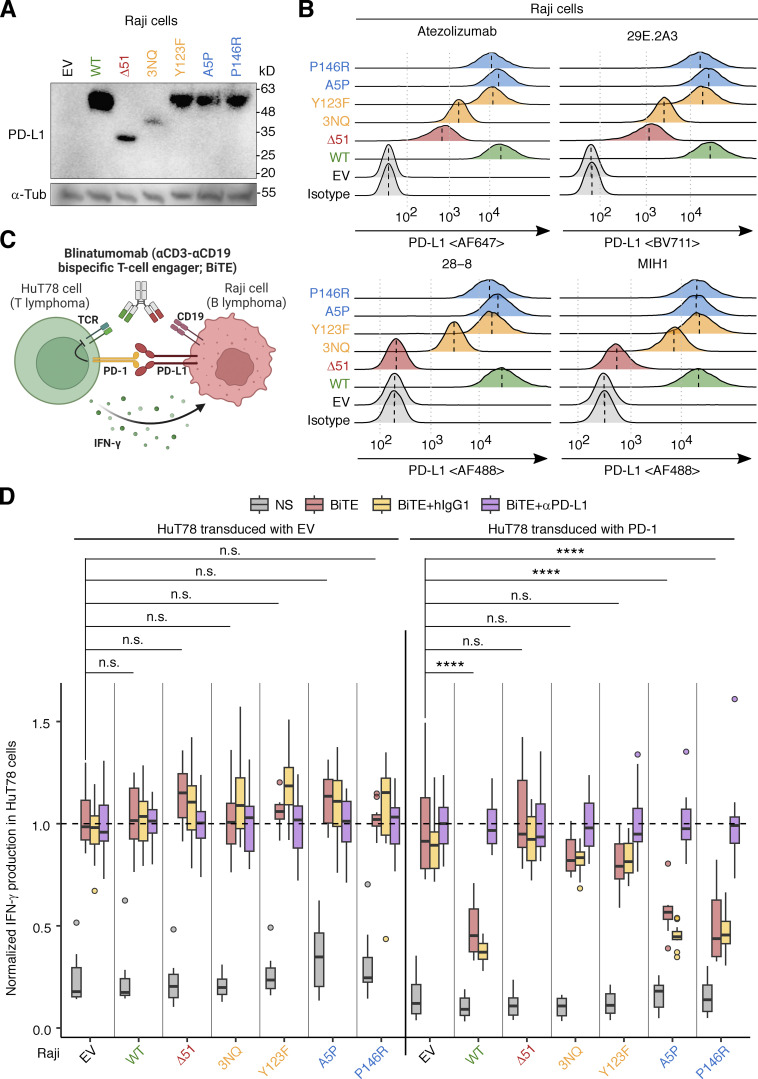
**Analysis of the PD-L1 protein with in-frame deletion in an overexpression system. (A and B)** PD-L1 protein levels. Raji B-lymphoma cells were lentivirally transduced with cDNA encoding the WT or a mutant PD-L1 isoform, or with EV, and were then subjected to selection on puromycin. PD-L1 protein levels were determined by (A) immunoblotting and (B) flow cytometry with monoclonal antibodies (mAb) against PD-L1. In B, a vertical dotted line within a histogram indicates the median. Representative results from two experiments are shown. **(C and D)** PD-1:PD-L1-mediated suppression assay. **(C)** Schematic diagram. HuT78 T-lymphoma cells lentivirally transduced with EV or with WT PD-1 were cocultured with Raji cells transduced with EV or a WT or mutant PD-L1 isoform for 24 h without stimulation or with blinatumomab (CD3-CD19 bispecific antibody, BiTE). Secretion inhibitors were added for the last 6 h. IFN-γ production was quantified by intracellular flow cytometry. The effect of anti-PD-L1 neutralizing mAb (equivalent to atezolizumab) or its isotype control was also assessed in this system. **(D)** Summary plot. The readout (percentage of IFN-γ^+^ HuT78 cells) was normalized against the mean in the “BiTE plus anti-PD-L1 antibody” group. Results from two independent experiments with 12 technical replicates in total were compiled. Statistical significance was determined for differences between EV and each PD-L1 construct in BiTE-stimulated conditions by two-tailed Wilcoxon’s rank sum tests with FDR adjustment. n.s., not significant. ****, P < 0.0001. Source data are available for this figure: SourceData F3.

The putative PD-L1 protein encoded by the NM_014143:r.530_682del transcript was predicted to have a 51-amino acid deletion in the extracellular domain (NP_054862.1:p.Gly177_Pro227del; referred to hereafter as PD-L1_∆51_) (Fig. 2 C). The deleted region overlaps the Ig-like C2 domain of PD-L1 (residues 133–225) but not the Ig-like V domain (residues 19–127) that interacts with PD-1 (Lin et al., 2008). We assessed the expression of the WT PD-L1 and PD-L1_∆51_ proteins in an overexpression system in which Raji B-lymphoma cells were transduced with a cDNA encoding the corresponding protein. The 51-amino acid deleted region contains three asparagine residues known to be glycosylated. The replacement of these residues with glutamine (PD-L1_3NQ_) results in lower levels of the protein due to enhanced proteasomal degradation (Li et al., 2016). We, therefore, also assessed the levels of PD-L1_3NQ_. Moreover, we also tested Y123F, as the Y123 residue is predicted to be structurally critical for the interaction between PD-1 and PD-L1 (Zak et al., 2017). Finally, we tested A5P and P146R, the only two variants present in the homozygous state in the gnomAD database. Immunoblotting analyses with an anti-PD-L1 mAb (clone E1L3N) showed that both PD-L1_∆51_ and PD-L1_3NQ_ were expressed in smaller amounts than WT PD-L1, whereas the other three missense variants were expressed in similar amounts to the WT protein (Fig. 3 A). Moreover, flow cytometry with two anti-PD-L1 mAbs (atezolizumab biosimilar and the 29E.2A3 clone) showed that PD-L1_∆51_ and PD-L1_3NQ_ were expressed at a level intermediate between the background and WT PD-L1 levels (Fig. 3 B). By contrast, another two anti-PD-L1 mAbs (clones 28-8 and MIH1) did not recognize PD-L1_∆51_ (Fig. 3 B). Again, the three missense variants were expressed in similar amounts to the WT protein (Fig. 3, A and B). These data suggest that the PD-L1_∆51_ isoform is inefficiently produced and translocated to the cell surface.

### The alternative PD-L1 protein isoform is loss-of-function in an overexpression system

Atezolizumab recognizes an epitope overlapping the interface for interaction with PD-1, whereas clone 29E.2A3 recognizes an epitope competing with CD80 (Córdova-Bahena and Velasco-Velázquez, 2020; Haile et al., 2013). The recognition of the PD-L1_∆51_ isoform by atezolizumab raised the question as to whether PD-L1_∆51_ retained the capacity to trigger the coinhibitory signaling of PD-1. We, thus, performed a coculture assay with EV- or PD-1-transduced HuT78 T-lymphoma cells and EV- or PD-L1-transduced Raji B-lymphoma cells to evaluate the suppressive function of the WT and mutant PD-L1 proteins in the presence of PD-1 (Fig. 3 C) (Ogishi et al., 2021). Cells were left unstimulated or were stimulated with blinatumomab, a CD3-CD19 bispecific antibody construct, and secretion inhibitors. We also tested an anti-PD-L1 neutralizing antibody (equivalent to atezolizumab) and a human IgG1 isotype control with an engineered constant region (N298A). We found that Raji cells expressing WT PD-L1 suppressed the activation of PD-1-transduced HuT78, as shown by flow cytometry assessments of IFN-γ production, whereas they did not suppress the activation of EV-transduced HuT78 cells (Fig. 3 D). This suppression was reversed in the presence of anti-PD-L1 antibody but not isotype control. In this system, the expression of PD-L1_∆51_, PD-L1_3NQ_, or Y123F on Raji cells led to no significant suppression of IFN-γ production by PD-1-transduced HuT78, indicating a complete loss-of-function (LOF). By contrast, A5P and P146R were functionally neutral. Thus, the patients’ *CD274* allele is completely LOF in an overexpression system due to the exclusive expression of the LOF PD-L1_∆51_ protein isoform. The observation that the PD-L1_∆51_ protein isoform is LOF despite its recognition by atezolizumab indicates that a conformational change due to the 51-amino acid deletion, rather than a disruption of the PD-1-interacting interface itself, is responsible for the lack of functionality.

### Exclusive expression of *CD274* mRNA with an in-frame deletion in the patients’ leukocytes

**Figure 4.**
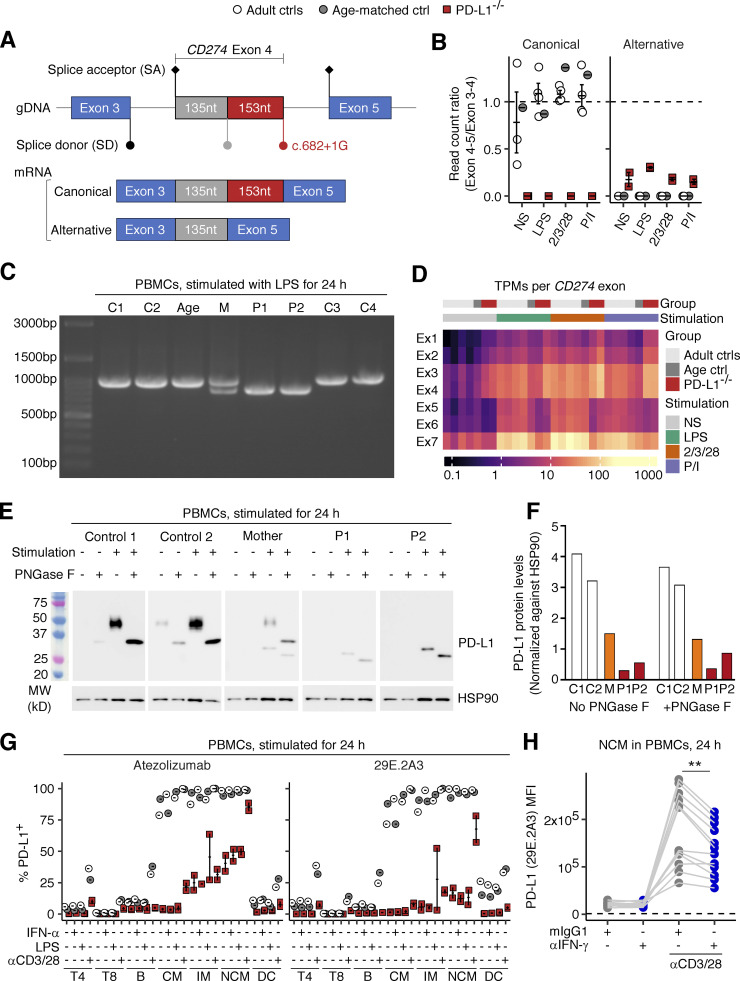
**Analysis of endogenously expressed *CD274* mRNA and PD-L1 protein in the patients’ leukocytes. (A, B, and D)** Bulk RNASeq analysis. PBMCs from the two PD-L1-deficient siblings (ages 11 and 10 years), and adult and age-matched controls were either left non-stimulated or were stimulated with lipopolysaccharide (LPS), anti-CD2/CD3/CD28 mAb cocktail, or phorbol 12-myristate 13-acetate and ionomycin (P/I) for 24 h. **(A)** A schematic diagram of the *CD274* mRNA exon 3-4-5 splice junctions in the cells of healthy donors (canonical) and the patients (alternative). **(B)** Ratio of read counts supporting the canonical and alternative exon 4–5 splice junction to read counts for the exon 3–4 splice junction. **(C)** RT-PCR products with a primer pair amplifying the whole *CD274* coding sequence derived from the total RNA of PBMCs stimulated with LPS for 24 h. **(D)** Expression levels (transcripts per million reads; TPM) for each *CD274* exon. **(E)** Western blot analysis for PD-L1 in PBMCs. PBMCs from the two PD-L1-deficient siblings (aged 11 and 10 years), their mother, and two healthy controls were either left non-stimulated or were stimulated with PHA overnight. Cell lysates were either left untreated or were treated with PNGase F, as indicated. **(F)** Densitometry results for the western blot shown in E. Values are normalized against the density of the loading control (HSP90). **(G)** Surface PD-L1 expression. PBMCs from the two PD-L1-deficient siblings (aged 11 and 10 years) and adult and age-matched controls were either left non-stimulated or were stimulated with IFN-α2, LPS, or anti-CD3/CD28 mAb-conjugated beads for 24 h. The level of PD-L1 expression on the surface of the cells of the different leukocyte subsets was determined by flow cytometry with two different mAbs against human PD-L1. **(H)** IFN-γ neutralization assay. PBMCs from healthy controls were either left non-stimulated or were stimulated with anti-CD3/CD28 mAb-conjugated beads for 24 h in the presence of anti-IFN-γ neutralizing mAb or its isotype control. PD-L1 levels were determined by flow cytometry with the 29E.2A3 clone. The horizontal dotted line indicates the level of background fluorescence determined with an isotype control for 29E.2A3. In B and G, bars represent the mean and SEM. In A–D and G, the experiments were performed once. In E and F, representative data from two experiments are shown. In H, results from three experiments (six donors in total) with technical duplicates are compiled. Statistical significance was determined for differences between IFN-γ neutralization and isotype control in anti-CD3/CD28-stimulated conditions by two-tailed paired Wilcoxon signed rank tests with FDR adjustment. **, P <0.01. Source data are available for this figure: SourceData F4.

**Figure S2.**
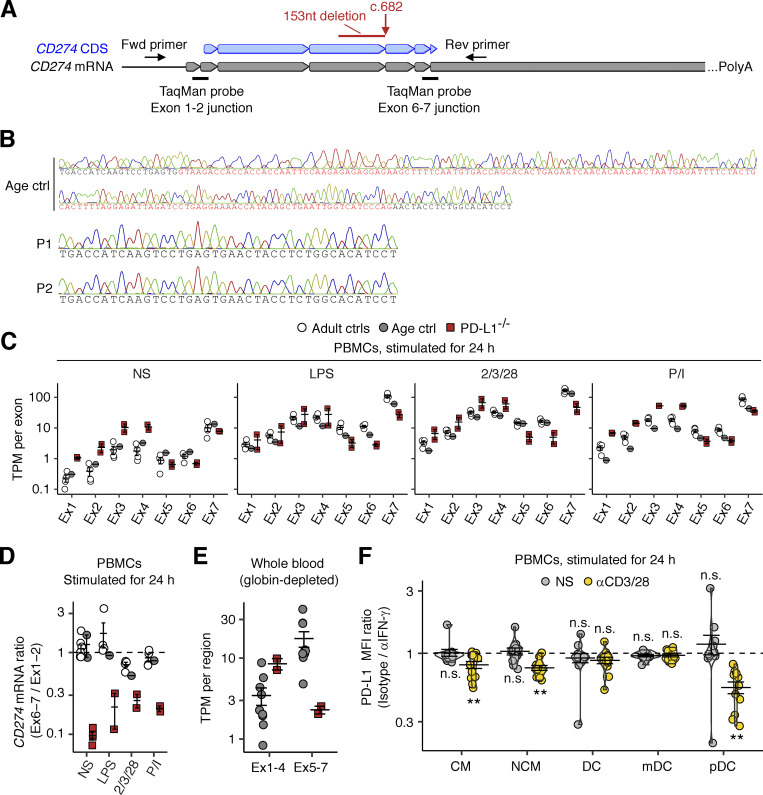
**Analysis of *CD274* mRNA in the patients’ leukocytes. (A)** Schematic diagram of the design of RT-PCR primers and TaqMan probes. **(B)** RT-PCR on the *CD274* CDS from total RNA extracted from PBMCs stimulated with LPS for 24 h. The 153nt deletion observed in bulk RNASeq data was confirmed by Sanger sequencing. **(C)** Bulk RNASeq for stimulated PBMCs. PBMCs from the two PD-L1-deficient siblings (aged 11 and 10 years), an age-matched control, and several healthy adult controls were either left unstimulated or were stimulated with LPS, anti-CD2/3/28 mAb cocktail, or PMA/ionomycin (P/I) for 24 h. Total RNA was used to prepare libraries for bulk RNASeq. TPM is shown for each *CD274* exon. **(D)** Quantitative PCR on the cDNA derived from the total RNA extracted from the LPS-stimulated PBMCs analyzed in C. *GUSB* was used as an endogenous control. Ratios of results for two TaqMab probes, targeting the exon 1–2 or 6–7 junction, are shown. The non-stimulated conditions were analyzed twice (technical replicates). **(E)** Bulk RNASeq on whole-blood leukocytes. Freshly drawn venous blood samples from the two PD-L1-deficient siblings (aged 11 and 10 years) and age-matched controls were used for total RNA extraction. Globin-depleted total RNA was used for sequencing. TPMs per region (*CD274* exons 1–4 and 5–7) are shown. **(F)** IFN-γ neutralization assay. PBMCs from healthy controls were either left non-stimulated or were stimulated with anti-CD3/CD28 mAb-conjugated beads for 24 h in the presence of anti-IFN-γ neutralizing mAb or its isotype control. PD-L1 levels were determined by flow cytometry with the 29E.2A3 clone. The fold-change decrease in PD-L1 MFI was calculated. In A–E, the experiments were performed once. In F, results from three experiments (six donors in total) with technical duplicates are compiled. The statistical significance of the difference between anti-IFN-γ and isotype control treatments was determined for each set of conditions in two-tailed Wilcoxon’s signed-rank tests with FDR adjustment. n.s., not significant. **, P < 0.01.

Our analyses up to this point suggested that the patients’ *CD274* allele was LOF in an overexpression system. We next asked if the patients’ *CD274* allele results in the same alternative splicing in the patients’ primary cells. To test this, we first performed bulk RNA sequencing (RNASeq) to analyze SD site use between exons 4 and 5 in peripheral blood mononuclear cells (PBMCs) from P1 and P2, their heterozygous mother (age 29 years), and healthy controls after treatment with different stimuli for 24 h (see Materials and methods). Consistent with the results for the overexpression system, all the reads for the patients’ cells were mapped to an alternative exon splice junction corresponding to the NM_014143:r.530_682del transcript (Fig. 4, A and B). To validate this finding, we performed RT-PCR on the same total RNA samples with a primer pair amplifying the complete *CD274* CDS (Fig. S2 A). Agarose gel electrophoresis and Sanger sequencing validated the presence of the canonical *CD274* transcript in five healthy controls, the NM_014143:r.530_682del transcript in the two patients, and both transcripts in the heterozygous mother (Fig. 4 C and Fig. S2 B). We noticed that the ratio of read counts for the alternative (NM_014143:r.530_682del) exon 4–5 splice junction to read counts for the exon 3–4 splice junction in the patients’ cells was much lower than that for the canonical exon 4–5 splice junction in control cells, suggesting that the RNA polymerase progressed inefficiently from exon 4 to exon 5 (Fig. 4 B). We therefore analyzed the levels of *CD274* mRNA per exon. Indeed, the patients’ cells had two to four times more read counts mapping to exons 1–4 than healthy controls but fewer read counts for exons 5–7 (Fig. 4 D and Fig. S2 C). Moreover, RT-qPCR on the same total RNA samples with two TaqMan probes targeting the exon 1–2 and 6–7 splice junctions (Fig. S2 A) validated the relative depletion of the exon 6–7 junction in the patients’ cells (Fig. S2 D). Finally, a similar pattern was observed in whole-blood leukocyte samples at baseline via RNASeq (Fig. S2 E). Overall, the patients’ leukocytes exclusively expressed the *CD274* mRNA isoform with the 153-base deletion and would therefore be expected to exclusively express the PD-L1_∆51_ protein isoform.

### Exclusive expression of the PD-L1_∆51_ protein isoform in the patients’ leukocytes

We then characterized the PD-L1 protein expressed in the patients’ primary leukocytes by analyzing lysates of PBMCs, either left unstimulated or stimulated with PHA overnight, by immunoblotting with an anti-PD-L1 mAb (clone 73–10). As the 51-amino acid deleted region contains three asparagine residues known to be sites of glycosylation, we also analyzed lysates following treatment with recombinant glycosidase (peptide-N-glycosidase F; PNGase F) to remove all N-linked glycans. The calculated molecular weight of the WT PD-L1 protein was 33 kD. As expected, untreated lysates from healthy controls displayed heterogeneous staining over molecular weights of 45–50 kD, decreasing to ∼33 kD upon PNGase F treatment (Fig. 4 E), suggesting that the PD-L1 protein in leukocytes was heterogeneously glycosylated. By contrast, homogeneous staining of a band at about 30 kD was observed with untreated lysates from both patients, decreasing to ∼26 kD upon PNGase F treatment, corresponding to the predicted molecular weight of the PD-L1_∆51_ isoform (27 kD) (Fig. 4 E). The presence of a single band is consistent with exclusive expression of the alternative *CD274* mRNA isoform with the 153-nt in-frame deletion identified by RT-PCR and bulk RNASeq (Fig. 4, C and D). The bands obtained for the heterozygous mother were consistent with the expression of both the WT and ∆51 isoforms of PD-L1 (Fig. 4 E). Quantification of the PD-L1 bands by densitometry showed that the levels of this protein were >80% lower than normal in both P1 and P2 and ∼50% normal levels in the heterozygous mother (Fig. 4 F). These data suggest that the patients’ primary leukocytes express exclusively the LOF PD-L1_∆51_ protein isoform, which is produced in only small amounts.

### Impaired but not abolished surface expression of PD-L1 protein on the patients’ leukocyte subsets

We assessed the levels of PD-L1 protein on the cell surface by analyzing PBMCs from the two siblings and healthy controls either left unstimulated or stimulated with IFN-α, lipopolysaccharide (LPS), or anti-CD3/CD28 mAb-conjugated beads for 24 h. We performed flow cytometry with the two anti-PD-L1 mAbs recognizing the PD-L1_∆51_ protein isoform (atezolizumab biosimilar and clone 29E.2A3). In leukocytes from healthy controls, classical/intermediate/non-classical monocytes strongly expressed PD-L1 regardless of the stimulation conditions (Fig. 4 G). CD11c^+^ DCs expressed PD-L1 weakly at baseline and moderately upon stimulation with bead-immobilized anti-CD3/CD28 mAbs (Fig. 4 G). CD4^+^ T and B lymphocytes expressed PD-L1 at a very low level at baseline, increasing to moderate levels upon stimulation with bead-immobilized anti-CD3/CD28 mAbs (Fig. 4 G). CD8^+^ T lymphocytes barely expressed PD-L1 at baseline, but weak expression was detected upon stimulation with bead-immobilized anti-CD3/CD28 mAbs (Fig. 4 G). By contrast, the levels of PD-L1 expression on the patients’ leukocytes were very low in all subsets and conditions tested, but the expression was not entirely abolished (Fig. 4 G). In particular, stimulation with anti-CD3/CD28 mAb-conjugated beads induced a modest increase in PD-L1 expression in the intermediate and non-classical monocytes of the patients (Fig. 4 G). Following the stimulation of PBMCs from multiple healthy donors with anti-CD3/CD28 mAb-conjugated beads, an IFN-γ-neutralizing mAb reduced the induction of PD-L1 on classical and non-classical monocytes, and on pDCs, suggesting that this induction of PD-L1 is IFN-γ-dependent (Fig. 4 H and Fig. S2 F). These data suggest that the two affected siblings had AR PD-L1 deficiency due to the exclusive expression of the LOF PD-L1_∆51_ isoform on the cell surface rather than a complete loss of PD-L1 expression.

### Milder dysregulation of leukocyte development in inherited PD-L1 deficiency than in PD-1 deficiency

**Figure S3.**
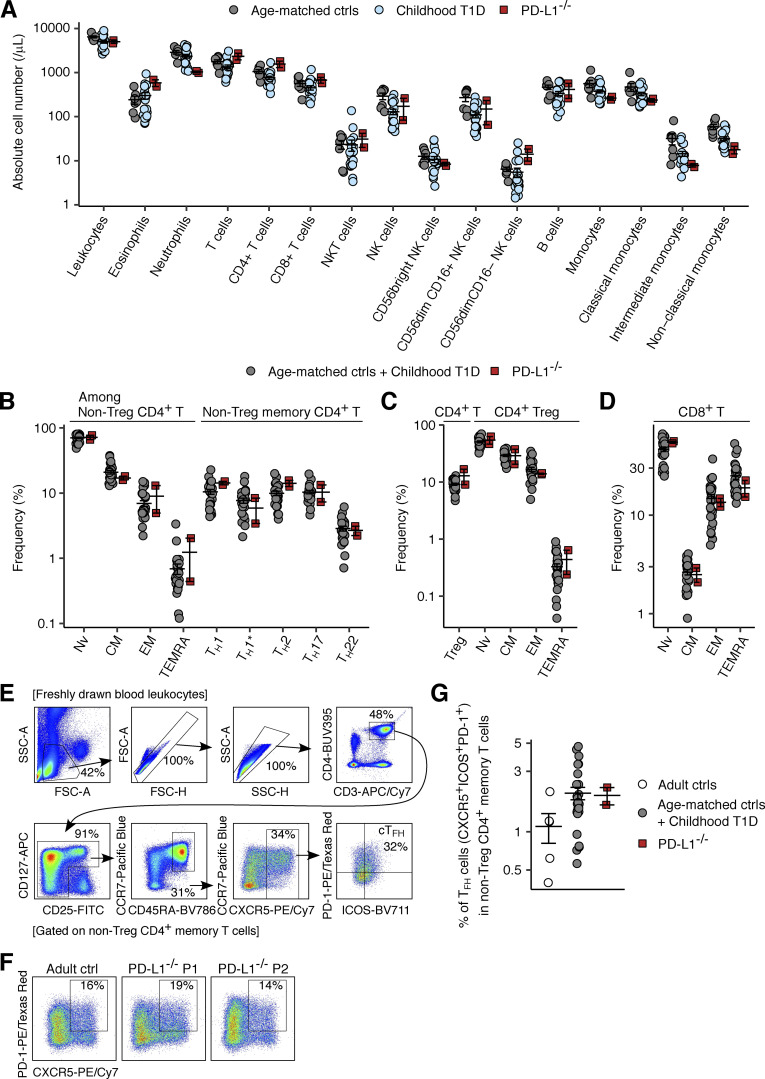
**Immunophenotyping analysis of PD-L1-deficient leukocytes.** Freshly drawn whole-blood leukocytes were analyzed by flow cytometry. **(A)** Absolute cell numbers were determined with Trucount Absolute Counting Tubes. **(B–D)** Frequencies of the given leukocyte subsets within each parental subset (indicated at the top of the plots). **(E)** Gating strategy for circulating T_FH_ (cT_FH_) cells. **(F)** Representative plots for cT_FH_ cells. **(G)** Percentage of cT_FH_ cells. In A–D and G, bars represent the mean and SEM.

**Figure 5.**
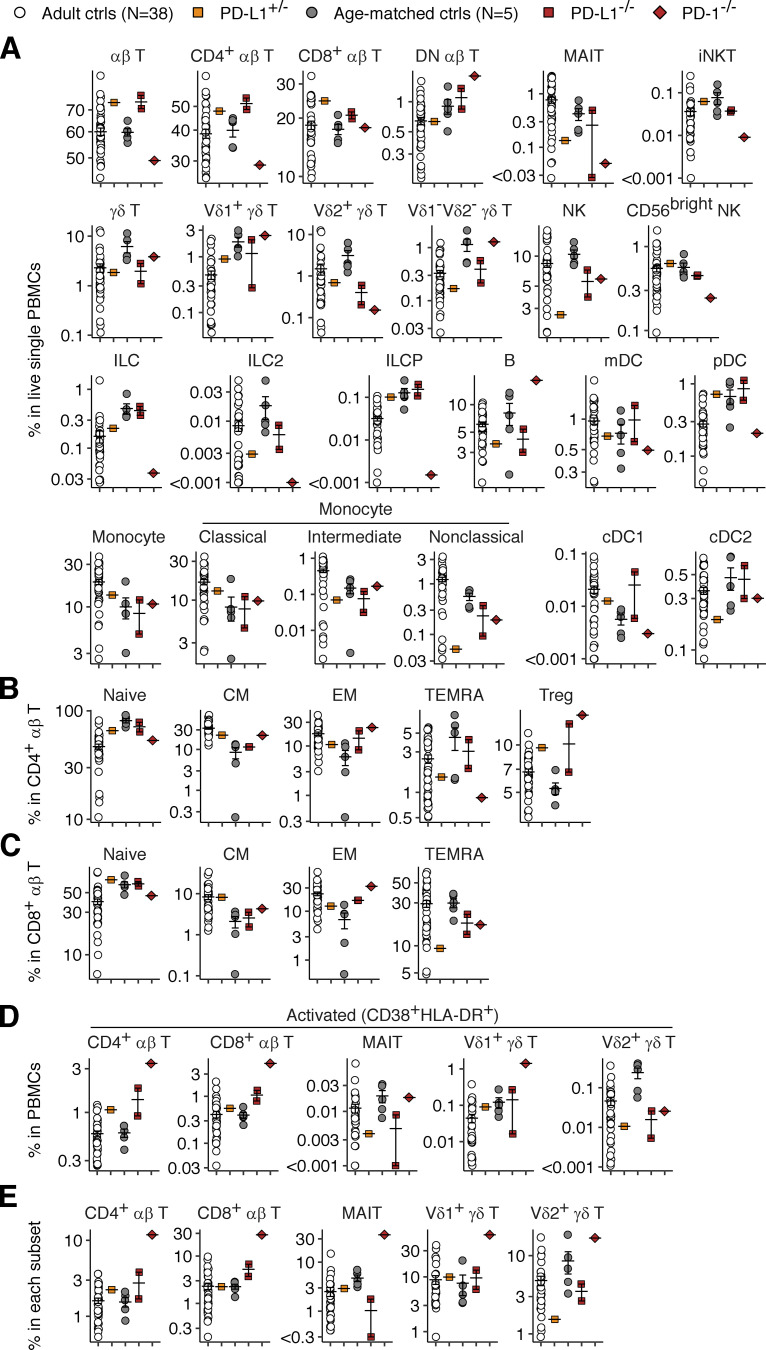
**Immunophenotyping analysis of PD-1- and PD-L1-deficient leukocytes.** Freshly thawed PBMCs from the two PD-L1-deficient siblings (aged 11 and 10 years), their mother, and adult and age-matched controls were immunophenotyped by flow cytometry. PBMCs from the previously described PD-1-deficient child (aged 11 years) were also analyzed with the same panel. **(A)** Proportions of leukocyte subsets in PBMCs. **(B)** CD4^+^ αβ T lymphocyte subsets. **(C)** CD8^+^ αβ T lymphocyte subsets. **(D and E)** Proportions of activated T lymphocyte subsets in (D) PBMCs and (E) each parental subset. Bars represent the mean and SEM.

We assessed the impact of PD-L1 deficiency on leukocyte development by analyzing the leukocytes in the freshly drawn venous blood samples by flow cytometry. However, we observed no marked differences in the absolute numbers of cells for the major lymphoid and myeloid leukocyte subsets between the two PD-L1-deficient siblings (sampled at ages 11 and 10 years) and healthy age-matched controls or childhood T1D controls (Fig. S3, A–E). We then performed deep immunophenotyping on PBMCs from the two PD-L1-deficient siblings, their heterozygous mother (aged 29 years), one pediatric control (11 years old), and one adult control (31 years old) by spectral flow cytometry (Fig. 5, A–E). The data were analyzed together with previously generated data from adult and pediatric controls. We also analyzed PBMCs from a previously described child with complete PD-1 deficiency (aged 11 years) (Ogishi et al., 2021) with the same panel. This PD-1-deficient child had smaller proportions of CD56^bright^ NK, Vδ2^+^ γδ T, and MAIT cells and an expansion of the CD4^−^CD8^−^ double-negative (DN) αβ cell population relative to age-matched controls, consistent with the previous characterization (Ogishi et al., 2021). Similarly, the proportions of Vδ2^+^ γδ T and NK lymphocytes in the two PD-L1-deficient siblings were smaller than those in the controls (Fig. 5 A). MAIT cells were also almost undetectable in one of the PD-L1-deficient siblings (Fig. 5 A). However, neither of these siblings presented a decrease in the proportion of CD56^bright^ NK cells or an expansion of the DN αβ T cell population. No marked alterations to the myeloid compartments were observed in the PD-1- and PD-L1-deficient patients. The T-lymphocyte subsets of the PD-1-deficient child displayed enhanced expression of CD38 and HLA-DR, the two surface markers typically expressed on activated T lymphocytes (Fig. 5, D and E). Notably, the CD4^+^ and CD8^+^ αβ T lymphocytes of the two PD-L1-deficient siblings also had levels of CD38 and HLA-DR expression moderately higher than those of age-matched controls but lower than those of the PD-1-deficient child. Thus, inherited PD-L1 deficiency triggers a dysregulation of leukocyte development milder than that in inherited PD-1 deficiency.

### Normal T cell receptor repertoire formation in inherited PD-L1 deficiency

**Figure 6.**
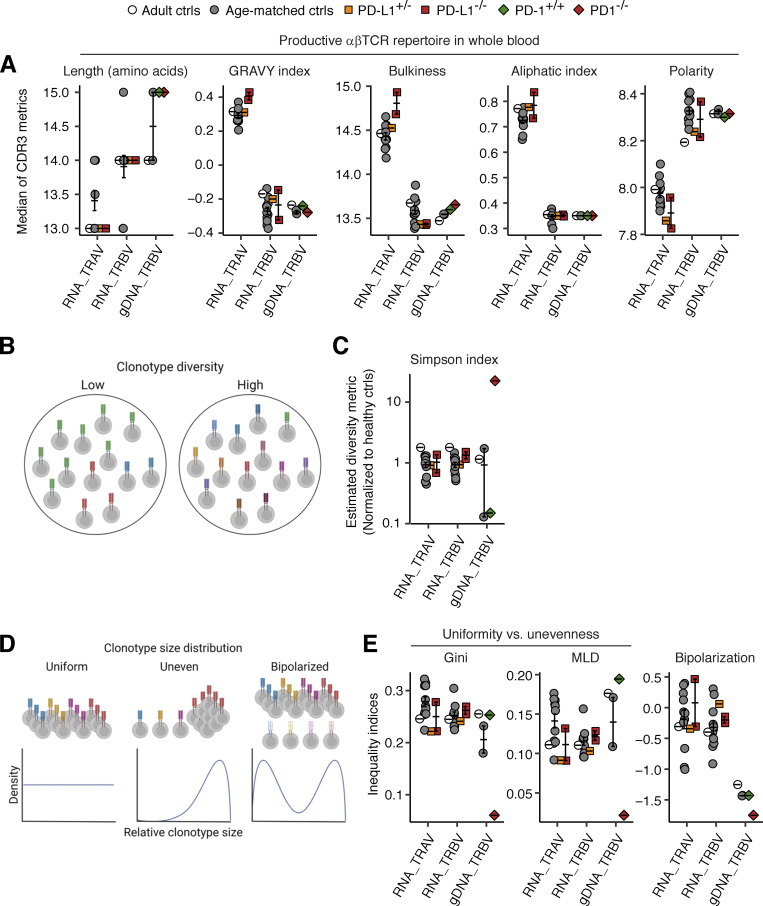
**Analysis of blood αβTCR repertoire in the PD-1- and PD-L1-deficient patients.** The complementarity-determining region 3 (CDR3) sequences in the *TRAV* and *TRBV* regions were reconstructed with MiXCR from bulk RNASeq datasets for whole-blood leukocytes from the two PD-L1-deficient siblings (aged 11 and 10 years), their mother, and adult and age-matched controls. For *TRBV*, the previously published Adaptive ImmunoSeq data for genomic DNA from the whole-blood leukocytes of the PD-1-deficient child (aged 10 years), his healthy brother (aged 6 years), and three healthy controls are also shown for comparison. **(A)** CDR3 length and physicochemical properties. The median values for each individual are shown. **(B and C)** CDR3 clonotype diversity. **(D and E)** Properties of the distribution of CDR3 clonotype sizes. Bars represent the mean and SEM.

We investigated whether inherited PD-L1 deficiency affected the formation of the T cell receptor (TCR) repertoire by analyzing the complementarity-determining region 3 (CDR3) sequences of the *TRAV* and *TRBV* regions reconstructed from bulk RNASeq datasets for whole-blood leukocytes from the two PD-L1-deficient siblings, their mother, healthy adults, and age-matched controls. We also performed a comparative reanalysis of the published *TRBV* CDR3 repertoire data derived from the genomic DNA of whole-blood leukocytes from the PD-1-deficient patient and his healthy brother, together with a healthy adult and age-matched controls (Ogishi et al., 2021). The estimated length and biochemical properties of the productive αβTCR CDR3 sequences were similar in healthy controls and the PD-L1-deficient siblings (Fig. 6 A). For *TRBV*, the findings were similar for healthy controls, PD-L1-deficient siblings, and the PD-1-deficient child, regardless of the source material used (Fig. 6 A). We then assessed the diversity of αβTCR CDR3 clonotypes. Rarefaction analysis showed that the PD-1-deficient child had more diverse clonotypes than healthy adults and age-matched controls, as measured by Simpson’s diversity index, whereas PD-L1-deficient siblings presented a diversity similar to that of healthy controls (Fig. 6, B and C). We then assessed the distribution of relative clonotype sizes in a given TCR repertoire. A completely uniform distribution is achieved when all identifiable clonotypes have exactly the same frequency in a given repertoire. A bipolarized distribution is achieved when a small number of clonotypes have frequencies much higher or much lower than the rest of the clonotypes. We used the Gini and mean-log deviation (MLD) indices to quantify uniformity (lower values indicate greater uniformity) and the Wolfson index to quantify bipolarization (lower values indicate a lesser degree of bipolarization). We found that the PD-1-deficient child had a more uniformly distributed and non-bipolarized clonotype size distribution than healthy controls and the PD-L1-deficient siblings (Fig. 6, D and E). These data suggest that inherited PD-L1 deficiency does not phenocopy inherited PD-1 deficiency, which alters peripheral TCR repertoire.

### Milder transcriptomic dysregulation in PD-L1-deficient than in PD-1-deficient leukocytes

**Figure 7.**
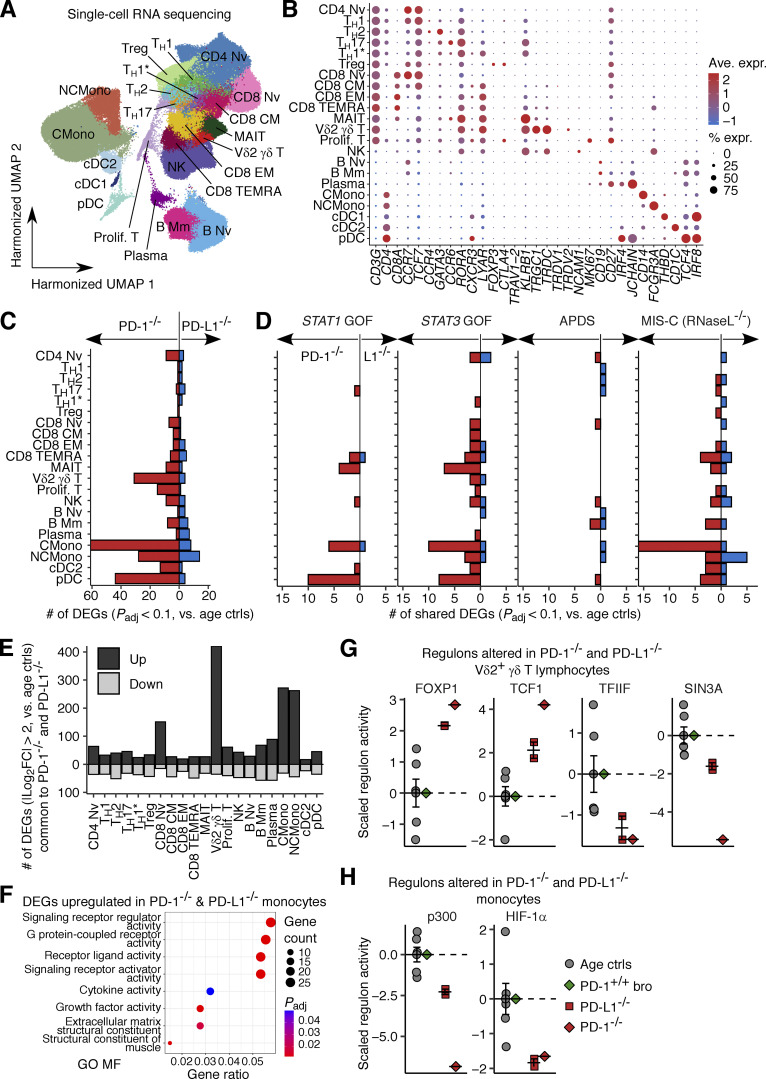
**Single-cell transcriptomic analysis of PD-1- and PD-L1-deficient leukocyte subsets.** scRNASeq was performed on cryopreserved PBMCs from the two PD-L1-deficient siblings (aged 11 and 10 years), their mother, and adult and age-matched controls. Previously generated datasets for healthy and diseased controls, including the PD-1-deficient child and his healthy brother, were also integrated into the analysis (Lee et al., 2023; Ogishi et al., 2021). **(A)** Clustering. Graph-based clustering was conducted after the removal of batch effects with Harmony (Korsunsky et al., 2019). Clusters were identified with SingleR (Aran et al., 2019) guided by the Monaco datasets (Monaco et al., 2019), followed by manual inspection. **(B)** Representative gene expression profiles. **(C)** Pseudobulk DE analysis. Individuals with PD-1 or PD-L1 deficiency were compared with age-matched controls, including the PD-1-deficient patient’s brother and an age-matched control for the PD-L1-deficient siblings. DE genes (DEGs) were defined as genes with FDR-adjusted P values <0.1. cDC1 was omitted because too few cells were captured for the PD-1-deficient patient. The numbers of DEGs per cell type are shown on a bar chart. **(D)** DEGs common to other monogenic etiologies of autoimmune or autoinflammatory disorders. For each condition, patients with monogenic disease were compared with age-matched controls. The numbers of DEGs (FDR-adjusted P value <0.1) common to (1) PD-1 or PD-L1 deficiency and (2) one of the four known monogenic forms of autoimmunity or autoinflammatory diseases are shown for each cell type. **(E)** DEGs common to the PD-1- and PD-L1-deficient leukocyte subsets. Here, DEGs are defined as genes with |log_2_FC| > 2 relative to age-matched controls. **(F)** Geneset overrepresentation analysis. DEGs upregulated in the classical or non-classical monocytes of PD-1- and PD-L1-deficient patients relative to age-matched controls were projected onto the Gene Ontology Molecular Function (GO MF) gene sets. GO MF gene sets for which significant enrichment was detected are shown. **(G and H)** SCENIC regulon activity analysis (Aibar et al., 2017) on (G) Vδ2^+^ γδ T cells and (H) monocytes (classical and non-classical combined). Single-cell regulon activities were aggregated to obtain a mean level of activity per cell type and per individual. The regulons most strongly differentially regulated in individuals with PD-1 and PD-L1 deficiencies relative to age-matched controls, as determined by two-tailed Wilcoxon’s rank sum test, are shown. Bars represent the mean and SEM.

**Figure S4.**
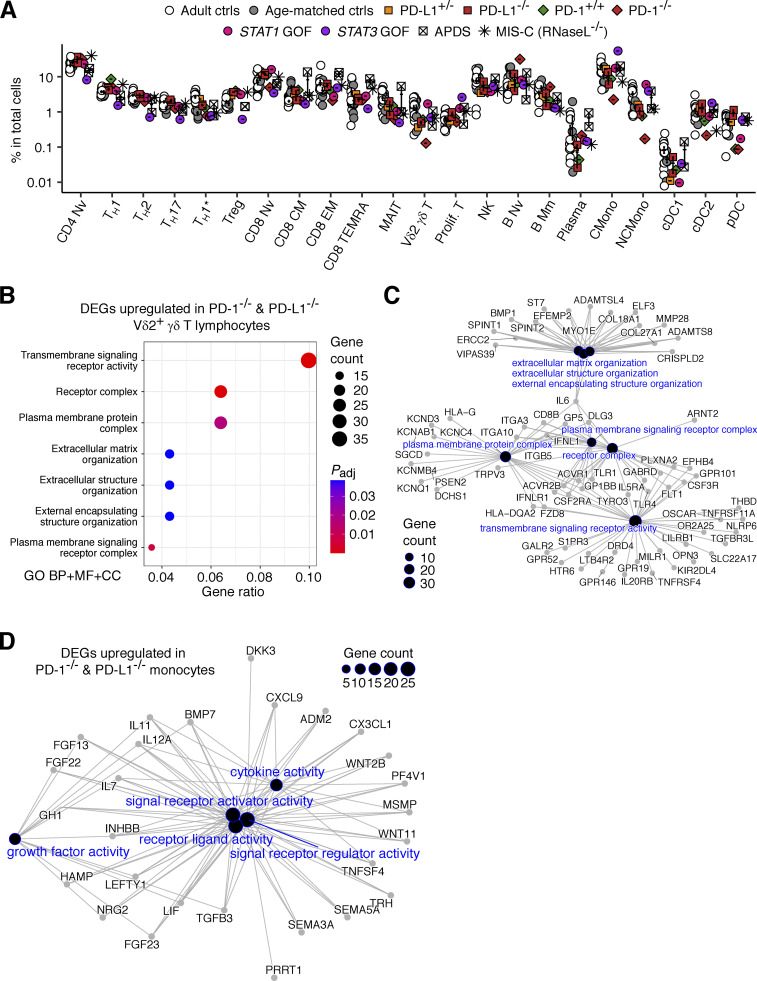
**Single-cell transcriptomic analysis.** scRNASeq was performed on cryopreserved PBMCs from the two PD-L1-deficient siblings (aged 11 and 10 years), their mother, and adult and age-matched controls. Previously generated datasets for healthy and diseased controls, including the PD-1-deficient child and his healthy brother, were also integrated into the analysis (Lee et al., 2023; Ogishi et al., 2021). Cell subsets were identified by unsupervised clustering followed by automated (i.e., SingleR) and manual annotation. **(A)** Frequencies of transcriptionally determined leukocyte subsets. **(B–D)** Pseudobulk DE analysis was performed to compare individuals with PD-1 or PD-L1 deficiency and age-matched controls, including the PD-1-deficient patient’s brother and an age-matched control for the PD-L1-deficient siblings. DE genes (DEGs) were defined as genes with |log_2_FC| > 2 relative to age-matched controls. **(B)** Geneset overrepresentation analysis. DEGs upregulated in Vδ2^+^ γδ T cells from PD-1- and PD-L1-deficient patients relative to age-matched controls were projected onto the gene ontology (GO) gene sets (BP for biological process, MF for molecular function, and CC for cellular component). GO gene sets for which significant enrichment was detected are shown. **(C and D)** Gene network plots for (C) Vδ2^+^ γδ T cells and (D) monocytes (classical and non-classical combined). DEGs contributing to a given GO term are connected by edges.

We explored more subtle phenotypic alterations in inherited PD-L1 deficiency by performing single-cell RNASeq (scRNASeq) analysis on PBMCs from the two PD-L1-deficient siblings, their mother, and both healthy adults and age-matched controls. For comparative analyses, we also included the data previously obtained for the PD-1-deficient patient and his healthy sibling (aged 10 and 6 years, respectively) (Ogishi et al., 2021), patients with a *STAT1* gain-of-function (GOF) mutation (*N* = 1), a *STAT3* GOF mutation (*N* = 1), activated PI3K delta syndrome (APDS; *N* = 2), and multisystem inflammatory syndrome in children due to RNaseL deficiency (MIS-C; *N* = 1) (Lee et al., 2023), together with seven healthy adult controls and six pediatric controls. Clustering analysis identified 22 distinct leukocyte subsets (Fig. 7, A and B). The proportions of transcriptionally defined leukocyte subsets in the PD-L1-deficient siblings were similar to those in healthy controls (Fig. S4 A), consistent with their globally normal immunophenotypes on flow cytometry (Fig. 5, A–C; and Fig. S3, A–D). Pseudobulk differential expression (DE) analysis revealed transcriptional alterations (DE genes with false discovery rate [FDR]–adjusted P values < 0.05) in multiple PD-1-deficient leukocyte subsets relative to age-matched controls (Fig. 7 C and Table S7). Moreover, of the four genetic etiologies of autoimmunity and autoinflammation (i.e., *STAT1* GOF, *STAT3* GOF, APDS, and MIS-C) analyzed simultaneously, *STAT3* GOF had the largest number of DE genes in common with PD-1 deficiency, suggesting a partial overlap in pathophysiology between these disease conditions (Fig. 7 D and Table S7). By contrast, PD-L1-deficient leukocytes had far fewer DE genes than their PD-1-deficient counterparts (Fig. 7, C and D; and Table S7). These data suggest that inherited PD-L1 deficiency underlies milder transcriptional dysregulation relevant to autoimmunity and autoinflammation in diverse leukocyte subsets in vivo than inherited PD-1 deficiency.

### Aberrant transcriptional signatures common to PD-1- and PD-L1-deficient leukocytes

We then assessed transcriptional signatures differentially up- or downregulated in PD-1- and PD-L1-deficient leukocytes relative to age-matched controls. The number of DE genes meeting the FDR-adjusted P value threshold in PD-L1-deficient leukocytes was small. We therefore instead considered all genes with absolute log_2_ fold-changes in expression >2. This analysis showed that the number of DE genes was greatest in Vδ2^+^ γδ T lymphocytes, followed by classical and non-classical monocytes and CD8^+^ naïve T lymphocytes (Fig. 7 E and Table S7). We observed a significant overrepresentation of genes involved in transmembrane signaling receptor activity, such as *TLR1*, *TLR4*, and *CSF3R*, genes commonly upregulated in PD-1- and PD-L1-deficient Vδ2^+^ γδ T lymphocytes (Fig. S4, B and C). Likewise, we found a significant overrepresentation of genes involved in receptor–ligand activity, such as *CXCL9*, *CX3CL1*, and *IL12A*, genes commonly upregulated in PD-1- and PD-L1-deficient monocytes (classical and non-classical combined) (Fig. 7 F and Fig. S4 D). We then used SCENIC (Aibar et al., 2017) to explore regulon activities altered in common PD-1- and PD-L1-deficient leukocyte subsets. In SCENIC, regulon activity is defined in every single cell on the basis of the enrichment in genes coexpressed with a gene encoding a given transcription factor (TF) and bearing a *cis*-regulatory motif for the TF. We aggregated each regulon activity by individual and leukocyte subset. We assessed 86 regulons and found that the activity of FOXP1 (encoded by *FOXP1*) and TCF1 (*TCF7*) was most enhanced, whereas that of TFIIF (*GTF2F1*) and SIN3A (*SIN3A*) was most reduced in PD-1- and PD-L1-deficient Vδ2^+^ γδ T lymphocytes (Fig. 7 G). Likewise, for the 163 regulons assessed, p300 (*EP300*) and HIF-1α (*HIF1A*) were the regulons displaying the greatest reduction in activity in PD-1- and PD-L1-deficient monocytes (Fig. 7 H). These results suggest that inherited PD-1 and PD-L1 deficiencies underlie overlapping transcriptomic alterations in T lymphocytes and myeloid cells in vivo despite the difference in clinical autoimmune manifestations and leukocyte development between these conditions.

### Impaired expression of IFN-γ by PD-L1-deficient leukocytes

**Figure 8.**
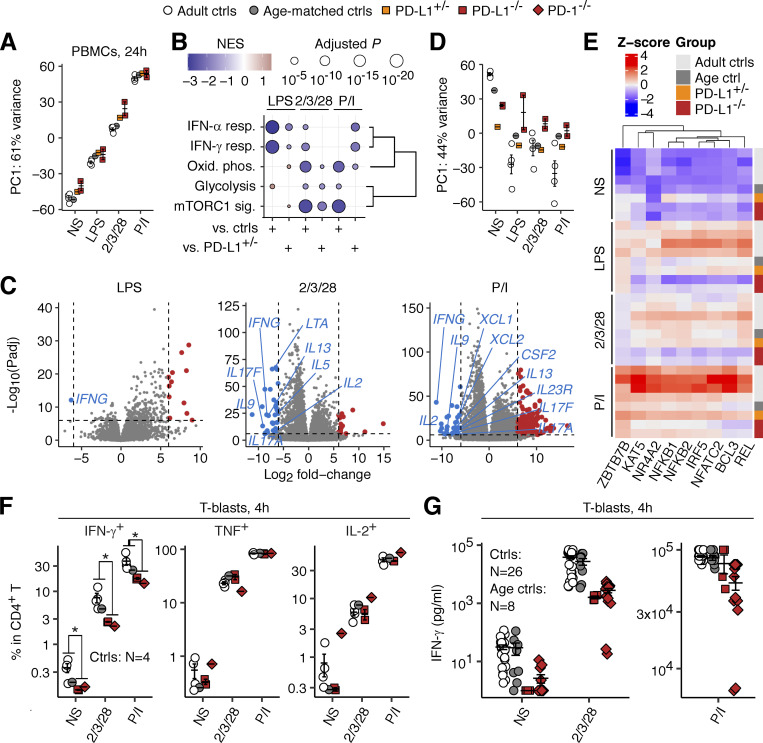
**Analysis of the cellular responses of PD-1- and PD-L1-deficient leukocytes in vitro. (A–E)** PBMC stimulation assay. PBMCs from the two PD-L1-deficient siblings (aged 11 and 10 years), their mother, and adult and age-matched controls were either left non-stimulated or were stimulated for 24 h. Bulk RNASeq was performed. **(A)** PCA. **(B)** GSEA. Genes were ranked based on their fold-change induction (stimulated versus non-stimulated) in PD-L1-deficient cells relative to either healthy controls or the heterozygous mother. Only significant results (FDR-adjusted P value <0.05) from 50 Hallmark gene sets are shown. Gene sets were reordered by hierarchical clustering for visualization purposes. **(C)** Differential gene induction. Genes related to cytokines or their receptors downregulated in PD-L1-deficient cells relative to control cells are labeled. **(D and E)** Transcription factor (TF) activity inference analysis based on the CollecTRI gene regulatory network database. **(D)** PCA. **(E)** Scaled activity for the TFs known to regulate *IFNG* mRNA levels lying in the top 30 for the loading of PC1 in D. **(F and G)** T-blast stimulation assay. **(F)** T-blasts were stimulated for 4 h in the presence of secretion inhibitors. Cytokine production was quantified by intracellular flow cytometry. Technical duplicates were prepared. **(G)** T-blasts were stimulated for 4 h, and cytokine secretion was quantified by multiplex ELISA. The age-matched controls include the healthy brother of the PD-1-deficient child. In A–E, the experiments were performed once. In F, representative data from two experiments are shown. In G, data from three experiments with technical replicates for PD-1-deficient cells (14 replicates for *N* = 1) and PD-L1-deficient cells (duplicates for *N* = 2) are compiled. In A, D, F, and G, the bars represent the mean and SEM. In F, statistical significance was determined for differences between all healthy controls combined and the PD-1/PD-L1-deficient patients combined by two-tailed Wilcoxon’s rank sum tests with FDR adjustment. *, P < 0.05.

**Figure S5.**
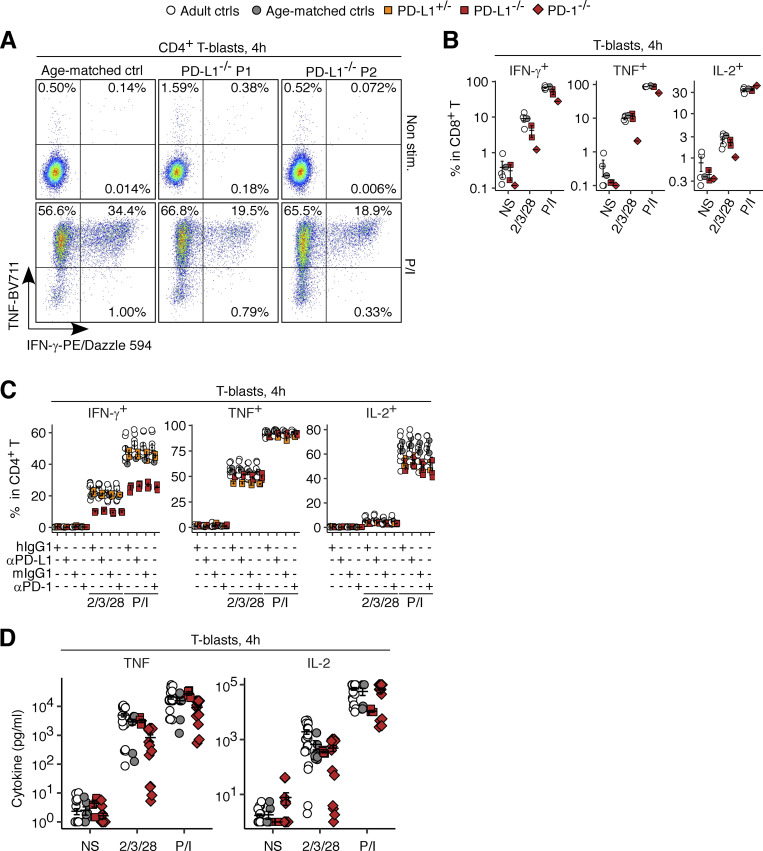
**Analysis of the cellular responses of PD-1- and PD-L1-deficient T lymphocytes in vitro.** T-blasts from healthy donors, a PD-1-deficient patient, and the PD-L1-deficient siblings and their heterozygous mother were either left non-stimulated or were stimulated with anti-CD2/CD3/CD28 mAb cocktail or PMA/ionomycin (P/I) for 4 h. **(A–C)** Intracellular cytokine levels were measured by flow cytometry. **(D)** Secreted cytokine levels were measured by multiplex ELISA. In B–D, bars represent the mean and SEM. In B, representative data from two experiments are shown. In C, the experiment was performed once. In D, data from three experiments with technical replicates for PD-1-deficient cells (14 replicates for *N* = 1) and PD-L1-deficient cells (duplicates for *N* = 2) are compiled.

Mouse PD-L1 deficiency results in higher levels of IFN-γ production by T cells, which has been thought to contribute to the autoimmunity-prone phenotype of these mice (Latchman et al., 2004). We, therefore, studied the transcriptional responses of human PD-L1-deficient leukocytes to external stimuli. We first analyzed bulk RNASeq data for PD-L1-deficient PBMCs left non-stimulated or stimulated with LPS, anti-CD2/3/28 mAb cocktail, or P/I for 24 h. Despite the globally normal transcriptional profiles of PD-L1-deficient leukocytes, as captured by PCA (Fig. 8 A), gene set enrichment analysis (GSEA) revealed significant impairment of the induction of genes known to be induced by IFN-γ (Fig. 8 B). *IFNG* was among the genes most significantly downregulated following stimulation with LPS, anti-CD2/CD3/CD28 mAbs, or P/I in PD-L1-deficient leukocytes relative to cells from healthy controls (Fig. 8 C). We then sought to identify the TF responsible for the observed defect of *IFNG* mRNA production. TF activity inference analysis predicted low levels of activity for *IFNG*-regulating TFs, including IRF5, NFAT1, NF-κB1/2, and c-Rel, in PD-L1-deficient leukocytes (Fig. 8, D and E). We then used flow cytometry to investigate cytokine production by an expanded T cell blast (T-blast) population stimulated with anti-CD2/CD3/CD28 antibodies or with P/I for 4 h. PD-1- and PD-L1-deficient CD4^+^ T-blasts had impaired IFN-γ production but normal levels of production for TNF and IL-2 in response to both stimuli (Fig. 8 F and Fig. S5 A). By contrast, PD-1-deficient CD8^+^ T-blasts displayed impaired IFN-γ, TNF, and IL-2 production upon stimulation with anti-CD2/CD3/CD28 antibodies, whereas no such defect was detected in PD-L1-deficient CD8^+^ T-blasts (Fig. S5 B). In this system, the blockade of PD-1 or PD-L1 with neutralizing mAbs in vitro did not affect cytokine production by either control or PD-L1-deficient cells, suggesting that PD-L1 deficiency impairs the development of IFN-γ-producing CD4^+^ T cells (Fig. S5 C). Finally, PD-1- and PD-L1-deficient T-blasts presented similar impairments of IFN-γ secretion following stimulation with anti-CD2/CD3/CD28 antibodies (Fig. 8 G). By contrast, only PD-1-deficient cells displayed a modest impairment upon stimulation with P/I, suggesting a milder phenotype in the PD-L1-deficient cells (Fig. 8 G). This poor IFN-γ production probably contributed to the severe TB observed in one of the two PD-1-deficient patients (Ogishi et al., 2021). The secretion of TNF was mildly impaired in PD-1-deficient, but not PD-L1-deficient, cells, whereas the secretion of IL-2 was not affected in either genotype (Fig. S5 D). Overall, inherited PD-L1 deficiency causes a mild impairment of IFN-γ production by T lymphocytes similar to that observed in inherited PD-1 deficiency.

## Discussion

We describe two siblings with inherited complete PD-L1 deficiency. These siblings have almost normal leukocyte development, suggesting a surprising level of redundancy of PD-L1 in humans. This contrasts strongly with the previous description of individuals with inherited PD-1 deficiency, which underlies an extensive dysregulation of both lymphoid and myeloid leukocyte subsets, including an expansion of the CD4^−^CD8^−^ DN αβ T-lymphocyte population and a decrease in the proportions of both Vδ2^+^ γδ T and MAIT cells (Ogishi et al., 2021). An expansion of the CD4^−^CD8^−^ DN αβ T-lymphocyte population is also seen in PD-1-deficient mice and patients treated with PD-1 blockade monotherapy (Ogishi et al., 2021). The apparent lack of leukocyte dysregulation in PD-L1-deficient patients can probably be attributed to the presence of inhibitory PD-L2:PD-1 signals. Alternatively, but much less likely, residual “leaky” expression of WT PD-L1 in vivo in certain cell types may also explain the relatively minor dysregulation of leukocytes in the two reported siblings. Finally, in light of recent findings that PD-L1 on antigen-presenting cells protects CD80 from CTLA-4-mediated trans-endocytosis and degradation through interaction with CD80 in *cis* (Sugiura et al., 2019; Zhao et al., 2019), it is also possible that PD-1 deficiency results in excess free PD-L1, potentially preventing the CTLA-4-mediated degradation of CD80 and, therefore, inducing much stronger CD80:CD28 costimulatory signals. By contrast, PD-L1 deficiency would be expected to result in less potent CD80:CD28 costimulatory signals due to the normal CTLA-4-mediated degradation of CD80 in this context. Further investigations are required to define more precisely the molecular mechanisms underlying the concordant and discordant clinical and immunological phenotypes in humans with PD-1 and PD-L1 deficiencies.

The two PD-1-deficient siblings died of autoimmune pneumonitis before the age of 12 years, whereas the two PD-L1-deficient siblings are currently well (with relevant hormone replacement therapies) at the ages of 11 and 10 years. The incidence of severe (grade 3–5) immune-related adverse events is similar or slightly higher in patients on PD-1 blockade (nivolumab or pembrolizumab; up to 3.5%) than in those on PD-L1 blockade (atezolizumab; up to 0.7%) (Martins et al., 2019). A similar trend has also been reported for treatment-related deaths (up to 0.5%, 0.9%, and 0% in trials of nivolumab, pembrolizumab, and atezolizumab, respectively) (Martins et al., 2019). The limitations of these comparisons include: (1) the limited number and young age of the PD-1- or PD-L1-deficient siblings studied relative to the much older individuals undergoing PD-1 or PD-L1 blockade and (2) the difficulty comparing different clinical trials of the blockade of PD-1 or PD-L1. Nevertheless, there is an overall concordance between these observations that PD-1 deficiency or blockade can trigger more severe autoimmunity than PD-L1 deficiency or blockade, these findings being consistent with the massive leukocyte dysregulation seen in patients with inherited PD-1 deficiency but not in those with PD-L1 deficiency. These findings suggest that PD-L2 can prevent certain types of autoimmunity in humans. To test this hypothesis, an in-depth study of germline variants of *PDCD1LG2*, encoding PD-L2, in the rare patients presenting severe adverse events following PD-L1 blockade is warranted. A search for individuals with inherited PD-L2 deficiency, by means of forward and reverse genetics, is also needed. Such studies may provide further insights into the redundant and nonredundant roles of PD-L1 and PD-L2 in the maintenance of self-tolerance in humans.

Clinically, the PD-L1-deficient siblings and the previously reported PD-1-deficient siblings all presented early-onset T1D. PD-1:PD-L1 signals are thought to be indispensable for the prevention of T1D, whereas PD-1:PD-L2 signals are not, because (1) PD-1- and PD-L1-deficient NOD mice develop rapid-onset autoimmune diabetes, whereas PD-L2-deficient NOD mice do not (Keir et al., 2006; Wang et al., 2005); and (2) the antibody-mediated blockade of PD-1 or PD-L1, but not that of PD-L2 or CTLA-4, induces autoimmune diabetes in NOD mice (Ansari et al., 2003). Autoimmune diabetes is also commonly seen in cancer patients after therapeutic PD-1 or PD-L1 blockade (Quandt et al., 2020). With the aim of identifying the molecular mechanisms underlying early-onset T1D, we systematically analyzed the leukocytic phenotypes of the PD-1- and PD-L1-deficient patients. Unexpectedly, we observed a substantial impairment of IFN-γ production by both PD-1- and PD-L1-deficient T lymphocytes, suggesting that these deficiencies render patients prone to tuberculosis and other intra-macrophagic infections (Ogishi et al., 2021). Thus, contrary to the initial study of PD-L1-deficient mice reporting increases in the production of IFN-γ and severe autoimmunity (Latchman et al., 2004), IFN-γ is unlikely to be a principal driver of autoimmunity in the absence of PD-1 or PD-L1 in humans. Additional studies are warranted to characterize the mechanisms underlying impaired cytokine production by PD-1- and PD-L1-deficient human T lymphocytes, particularly for IFN-γ. Moreover, in addition to T cell defects, we have also shown that memory B cell formation and antibody responses are impaired in PD-1- and PD-L1-deficient patients and in mice deficient for PD-1 signaling (unpublished data). We show that PD-1:PD-L1 interaction on B cells in *cis* actually promotes the induction of c-Myc, a critical regulator of B cell class switching, differentiation, and proliferation (Fernández et al., 2013), and consequently enhances the production of IgG. In vitro PD-1 and PD-L1 blockade phenocopies some of the cellular defects seen in PD-1- and PD-L1-deficient patients. However, similar to the impairment of IFN-γ production, the weakened antibody response is unlikely to be responsible for the pathogenesis of early-onset T1D. Other than these two cellular phenotypes, which are common to both PD-1- and PD-L1-deficient patients, PD-L1 seems to be redundant in most of the leukocyte compartments studied.

Theoretically, autoimmune endocrinopathy may be caused largely by two mechanisms that are not mutually exclusive: self-reactive T cells and autoantibodies. One of the two PD-L1-deficient siblings and both PD-1-deficient siblings suffered from autoimmune hypothyroidism. The PD-L1-deficient proband tested positive for anti-TPO autoantibodies. Likewise, the blockade of PD-1 or PD-L1 triggers autoimmune thyroid dysfunction with a high prevalence of anti-thyroid autoantibodies (Osorio et al., 2017). By contrast, no pancreatic islet autoantibodies (directed against GAD and IA-2) were detected in clinical tests performed on the two PD-L1-deficient patients and the PD-1-deficient proband (Ogishi et al., 2021). PD-L1 is known to be strongly expressed by pancreatic β cells (Colli et al., 2018). The expression of PD-L1 by pancreatic β cells is, therefore, probably essential to prevent autoantigen-specific PD-1-expressing αβ T lymphocytes from invading and destroying the pancreatic islets. Nevertheless, it is also possible that other PD-L1-expressing cells in the pancreas, such as DCs, are indispensable for this protection. Indeed, the two recent cancer immunology papers have shown that DCs are a crucial source of PD-L1, despite being vastly outnumbered by PD-L1^+^ macrophages, for the repression of antitumor CD8^+^ T cell responses in mice (Oh et al., 2020; Peng et al., 2020). These findings imply that not all PD-L1-expressing cells are of equal pathophysiological significance. Further studies are therefore required to determine the precise molecular and cellular roles of PD-L1-expressing cells in preventing T1D in the context of inherited or acquired PD-1 or PD-L1 deficiency.

## Materials and methods

### Case report

The index case was a Moroccan boy born to second-degree consanguineous parents in 2010. He was referred for genetic testing (https://www.diabetesgenes.org) at the age of 8 mo following a diagnosis of diabetes 14 h postpartum, with a blood glucose concentration of 14.9 mmol/liter. He has since been treated with insulin at a full replacement dose (current dose 0.75 U/kg/day, HbA1c 6.2%). This patient had recurrent bronchiolitis in infancy, followed by asthma diagnosed at the age of 5 mo, and developed TPO antibody-positive autoimmune hypothyroidism at the age of 3 years (treated with *L*-thyroxine). He was diagnosed with growth hormone (GH) deficiency at the age of 10 years and is currently on GH replacement therapy (recombinant somatotropin, Genotonorm, 0.8 mg/day). The patient was diagnosed with epilepsy at the age of 6 years, following a seizure with an abnormal electroencephalogram but a normal brain MRI scan. He was subsequently treated with potassium valproate and has remained seizure-free. He also suffered from mild intellectual disability with some delay in language acquisition. His sister, born in 2011, was referred for testing at the age of 3 mo after presenting with diabetic ketoacidosis and a blood glucose concentration of 22.2 mmol/liter at the age of 7 wk. She has also since been treated with insulin at a full replacement dose (currently 1.23 U/kg/day, HbA1c 8.7%) and is otherwise healthy.

### Human subjects

Healthy volunteers were recruited at The Rockefeller University. The siblings, both of whom were homozygous for a *CD274* splice-site variant, and their parents were referred to the Genetic Beta Cell Research Bank at the University of Exeter, UK, for genetic testing to determine the cause of their neonatal-onset diabetes. Healthy controls matched with the PD-L1-deficient siblings for age were recruited at the Benaroya Research Institute in the United States. The PD-1-deficient patient (homozygous for c.105dupC, p.Thr36Hisfs*70 in *PDCD1*) and his healthy brother (Ogishi et al., 2021), the RNaseL-deficient MIS-C patient (homozygous for c.793G>T, p.Glu265* in *RNASEL*) (Lee et al., 2023), two APDS patients (heterozygous for c.3061G>A, p.Glu1021Lys in *PIK3CD* or c.1425+1G>T in *PIK3R1*), and one STAT3 GOF patient (heterozygous for c.1255G>C, p.Gly419Arg in *STAT3*) were recruited at the Necker Hospital for Sick Children. Written informed consent was obtained from all patients, family members, and healthy volunteers enrolled in this study. The study was approved by the North Wales Research Ethics Committee (22/WA/0268, IRAS project ID 316050) and the institutional ethics committees of The Rockefeller University, Necker Hospital for Sick Children, and the Benaroya Research Institute and was performed in accordance with the requirements of these bodies. Experiments on samples from human subjects were conducted in the United Kingdom and the United States, in accordance with local regulations and with the approval of the institutional review board of the corresponding institution.

### Sample collection and DNA extraction

Peripheral whole blood was collected in 2-ml collection tubes containing EDTA. Automated DNA extraction was performed on a Chemagic STAR (Hamilton Bonaduz) system with the Chemagic STAR DNA blood extraction kit (CMG-1756; PerkinElmer).

### WGS

WGS was performed on a BGISEQ-500 sequencing platform for the affected siblings and both their parents. The mean read depth across the whole genome was 34.8 and the mean coverage at 20× was 95.2% (full metrics given in Table S1). We used BWA-MEM version 0.7.15 to align the raw sequence data, which were then processed with a custom-built pipeline based on GATK best practices (Picard version 2.7.1, GATK version 3.7). Variants were annotated with Alamut Batch Standalone version 1.11. Copy number variants were called with SavvyCNV (Wakeling et al., 2019). The percent homozygosity was calculated with SavvyVcfHomozygosity (Wakeling et al., 2019). The percent contamination was determined with VerifyBamID (Jun et al., 2012). The *CD274* splice-site variant was validated by Sanger sequencing of the PCR product from gDNA.

The self-reported Moroccan ethnicity of the affected siblings and their family members was confirmed by a PCA of WGS variants. For an ancestry-level analysis, the Human Genome Diversity Project and 1,000 Genomes phase 3 global genomic reference population datasets (Auton et al., 2015; Bergström et al., 2020) were used as the reference. Genetic ancestry labels were taken from the gnomAD-hosted versions of the datasets. For a country-level analysis, the dataset generated by Henn et al. (2012) for individuals from various Southern European and African populations was used. Quality control was performed to filter out loci on the basis of missingness rate (<0.05), allele frequency (>0.05), and LD (R^2^ < 0.8). PCA was performed on the remaining ancestry-informative SNPs (*N* = 813,637 and 208,379, respectively) with PLINK2 (Chang et al., 2015). The resulting eigenvectors were used to generate the PCA plot.

For the analysis of variants in genes known to underlie IEI, gene lists were taken from Bousfiha et al.(2022) and the NHS PanelApp Primary immunodeficiency or monogenic inflammatory bowel disease (panel 398) “Green” genes (Martin et al., 2019). We retained 486 curated disease-causing genes in total for the analysis. Variants called in coding and flanking intronic regions were considered. All variants present in the ClinVar or HGMD databases were kept for further examination; otherwise, variants of minimum read depth 10× or higher, minimum mapping quality of 40 or higher, and GnomAD MAF < 0.1% were retained for further examination. No variants in genes known to underlie IEI were detected in the homozygous or compound heterozygous state in both P1 and P2. Six variants were detected in the heterozygous state in both patients (Table S3). Therefore, the siblings were potential carriers of genes underlying five AR IEI. They were heterozygous for a missense variant of *CFHR5*, for which mutations can cause a dominant disorder, namely, nephropathy due to CFHR5 deficiency (Martin et al., 2019). This congenital disorder is characterized by early-onset kidney failure due to membranoproliferative glomerulonephritis. However, neither of these siblings has ever displayed any manifestations of kidney disease (current ages 11 and 10 years), suggesting that the observed missense variant is benign.

For de novo variant analysis, the same filtering criteria described in the above paragraph were applied. Two de novo variants were detected in the male proband (P1) and three were detected in his affected sister (P2) (Table S4). The corresponding genes are not known to be linked to any IEI.

### Cells

PBMCs were isolated by the Ficoll-Hypaque density gradient centrifugation of venous blood samples and cryopreserved at −150°C until use. Thawed PBMCs were allowed to rest temporarily during experiments in RPMI-1640 medium with GlutaMAX supplemented with 10% FBS (lymphocyte medium). The HEK293T cell line was purchased from the ATCC and cultured in DMEM supplemented with 10% FBS. The HuT78 T-lymphoma and Raji B-lymphoma cell lines were purchased from the ATCC and cultured in lymphocyte medium. T-blasts were generated by culturing PBMCs in ImmunoCult XF T Cell Expansion Medium (STEMCELL Technologies) supplemented with recombinant human interleukin 2 (rhIL-2; Cat: 11147528001; Roche) at a final concentration of 10 ng/ml and ImmunoCult Human CD3/CD28/CD2 T Cell Activator (1:100; STEMCELL Technologies), as previously described (Ogishi et al., 2023).

### Exon trapping

Exon trapping was performed as previously described (Nisson et al., 1994). Briefly, a segment of exon 4 of a canonical *CD274* isoform, flanked by intronic regions (300 bp upstream and 282 bp downstream), was amplified from genomic DNA extracted from T-blasts from P1 and one healthy age-matched control. The fragment was inserted into the pSPL3 vector by in-fusion cloning. HEK293T cells were transfected with either the empty pSPL3 vector (EV) or vectors containing the WT or mutant *CD274* exon 4 region. Total RNA was extracted 24 h later and the spliced mRNA was amplified by RT-PCR with the SA2 and SD6 primers (Nisson et al., 1994). The amplicon was inserted into the pCR4-TOPO vector (Invitrogen) and used to transform Stellar competent cells (Clontech). PCR amplicons from colonies were then sequenced to investigate the splicing products transcribed from the WT and mutant alleles.

### Lentiviral transduction

A full-length WT human *PDCD1* or *CD274* coding sequence (CDS) inserted into a pTRIP-CMV-Puro-2A backbone (plasmid #102611; Addgene, a gift from Nicolas Manel [Gentili et al., 2015]) was prepared in a previous study for lentiviral transduction (Ogishi et al., 2021). For *CD274*, we constructed a CDS corresponding to the alternative NM_014143:r.530_682del transcript (NP_054862.1:p.Gly177_Pro227del), a variant with three asparagine residues known to be glycosylated replaced by glutamines (termed PD-L1_3NQ_) (Li et al., 2016), a variant with the tyrosine residue at position 123 predicted to be structurally critical for the interaction between PD-1 and PD-L1 replaced by phenylalanine (Y123F) (Zak et al., 2017), and the homozygous variants in the gnomAD database (A5P and P146R) through site-directed mutagenesis. The entire CDS was validated by Sanger sequencing.

Lentiviruses were prepared by transfecting HEK293T cells with the pTRIP-CMV-Puro-2A plasmid (EV) or the vector carrying the WT or mutant *CD274* CDS, together with helper plasmids, as previously described (Ogishi et al., 2021). Viral supernatants were concentrated with a Lenti-X Concentrator (Cat: 631232; Takara Bio), resuspended in lymphocyte medium, and used immediately for transduction. Viral supernatant (100 μl per well) was added to HuT78 or Raji cells resuspended in 100 μl lymphocyte medium. The cells were spinoculated for 2 h at 1,200 × *g* at 25°C and then incubated for 48 h at 37°C. Puromycin (Cat: ant-pr-1, 10 µg/ml; InvivoGen) was added 48–96 h after spinoculation.

### Analysis of PD-L1 expression

Lysates of lentivirally transduced Raji cells were analyzed by immunoblotting, as previously described (Ogishi et al., 2021), with monoclonal antibodies (mAbs) against human PD-L1 (1:1,000, Clone: E1L3N; Cell Signaling Technology, 4°C overnight) and α-tubulin (1:200, Clone: B-7; Santa Cruz Biotechnology, 4°C overnight). For surface PD-L1 expression, lentivirally transduced Raji cells were stained with one of the four anti-PD-L1 mAbs (eBioscience, MIH1, Alexa Fluor 488, 1:100; Abcam, 28-8, Alexa Fluor 488, 1:100; BioLegend, 29E.2A3, BV711, 1:100; R&D Systems, #Hu124, Alexa Fluor 647, 1:100) or isotype controls (eBioscience, mouse IgG1, P3.6.2.B.1, Alexa Fluor 488; Abcam, rabbit IgG, EPR25A, Alexa Fluor 488; BioLegend, mouse IgG2b, MPC-11, BV711; Novus Biologicals, human IgG1 polyclonal isotype control, Alexa Fluor 647) for 1 h at 4°C and analyzed via flow cytometry, as previously described (Ogishi et al., 2021).

PBMCs were either left non-stimulated or were stimulated with IFN-), LPS, PHA-L, or anti-CD3/CD28 mAb-conjugated beads for 24 h. For immunoblotting analysis, cells were lysed in 75 μl RIPA buffer (50 mM Tris-HCl [pH 7.4], 150 mM NaCl, 0.1% SDS, 0.5% sodium deoxycholate, 1% NP-40, 200 μM sodium orthovanadate, and protease inhibitors [Roche]). Protein lysates were denatured by incubation at 100°C for 10 min in glycoprotein denaturing buffer and were then deglycosylated by treatment for 1.5 h with Pngase F (NEB) according to the manufacturer’s instructions. Reactions were terminated by adding 6× Laemmli buffer. Cell lysates were then subjected to SDS-PAGE in 12% acrylamide gels and western blots were performed with rabbit antibodies against HSP90 (GeneTex) and PD-L1 (clone 73-10, Abcam). Blots were visualized by ImageQuant with anti-rabbit HRP-linked antibodies (Cell Signaling Technology). Densitometry analysis was performed with ImageJ. For flow cytometry analysis, cells were harvested, washed with FACS buffer (PBS supplemented with 2 mM EDTA and 0.2% BSA), surface-stained for lineage markers (anti-CD4-APC-H7, anti-CD8-BV605, anti-CD19-PE, anti-CD14-Alexa Fluor 488, anti-CD16-PE/Cy7, and anti-CD11c-BV421 antibodies) and with one of the two anti-PD-L1 mAbs (29E.2A3, APC; BioLegend; #Hu124, Alexa Fluor 647; R&D Systems) at room temperature in the dark for 30 min. They were then washed, stained with 7-amino-actinomycin D (7-AAD), and acquired. Data were analyzed with FlowJo v10 (FlowJo, LLC) and R software.

### Analysis of PD-L1 expression with IFN-γ blockade in vitro

PBMCs (1 × 10^5^ cells per well) were either left non-stimulated or were stimulated with anti-CD3/CD28 mAb-conjugated beads (11131D; Gibco; 1 × 10^5^ beads per well) for 24 h, together with anti-IFN-γ neutralizing mAb (NIB42, 10 μg/ml; eBioscience) or isotype control (MOPC-21, 10 μg/ml) in a 96-well U-bottom plate. The non-adherent cells were transferred to a 96-well V-bottom plate by pipetting. The residual adherent cells were detached by incubation with the TryPLE enzyme (Gibco) at 37°C for 5 min and were then transferred to the same 96-well V-bottom plate. Cells were washed once with FACS buffer, surface-stained by incubation at 4°C for 1 h with various antibodies (anti-CD3-FITC [UCHT1, 1:100; Cytek], anti-CD19-BV650 [HIB19, 1:100; BD], anti-CD56-V450 [B159, 1:50; BD], anti-CD14-Spark NIR 685 [63D3, 1:100; BioLegend], anti-CD16-PE/Dazzle 594 [3G8, 1:100; BioLegend], anti-HLA-DR-APC/Fire 810 [L243, 1:100; BioLegend], anti-CD123-BV480 [9F5, 1:100; BD], anti-CD11c-Alexa Fluor 700 [Bu15, 1:1,000; BioLegend], anti-PD-1-BB700 [EH12.1, 1:100, BD], and anti-PD-L1-BV711 [29E.2A3, 1:100; BioLegend] or an isotype control [MPC-11; BioLegend]), washed, stained with 7-AAD (1:200), and acquired with an Aurora cytometer (Cytek). Data were analyzed with FlowJo software. The median fluorescence intensity (MFI) of PD-L1 was used as a readout.

### HuT78 and Raji cell coculture assay

HuT78 cells (1 × 10^5^ cells per well) and Raji cells (1 × 10^5^ cells per well) were cocultured in lymphocyte medium for 24 h with or without anti-CD19-anti-CD3 bispecific antibody (equivalent to blinatumomab) (10 ng/ml; BPS Bioscience) and anti-PD-L1-hIgG1 antibody (N298A; equivalent to atezolizumab) (Cat: hpdl1-mab12, 5 μg/ml; InvivoGen) or isotype control (Cat: bgal-mab12; InvivoGen). Monensin and brefeldin A (1:1,000 each; Cytek) were added for the last 6 h. Cells were stained with Zombie NIR Fixable Viability dye (1:1,000 in PBS; BioLegend) for 15 min at 4°C in the dark, washed with FACS buffer, and fixed and permeabilized with the Foxp3/Transcription Factor Staining Buffer Set (Cytek). Cells were then stained by incubation overnight at 4°C in the dark with the following reagents in permeabilization buffer: FcR blocking reagent (1:50; Miltenyi Biotec), anti-CD3-APC (Clone: UCHT1, 1:100; Cytek), anti-IFN-γ-PE-Dazzle 594 (Clone: 4S.B3, 1:500; BioLegend), and anti-TNF-BV711 (Clone: MAb11, 1:500; BioLegend) mAbs. The cells were washed with FACS buffer and acquired with an Attune NxT Flow Cytometer with the CytKick MAX Autosampler (Invitrogen). Data were analyzed with FlowJo and R software. The percentage of IFN-γ^+^ cells was used as a readout. The data were normalized against the mean for the blinatumomab plus atezolizumab group for each combination of HuT78 and Raji cells.

### Bulk RNASeq analysis of stimulated PBMCs

PBMCs (1 × 10^5^ cells per well) were either left non-stimulated or were stimulated with LPS (Cat: L4641-1MG, 10 ng/ml; Sigma-Aldrich), ImmunoCult Human CD3/CD28/CD2 T Cell Activator (1:100; STEMCELL Technologies), or Cell Stimulation Cocktail (Cat: 00-4970-93, 1:1,000; eBioscience) for 24 h. The cells were then collected by centrifugation, the cell pellets were lysed with DNA/RNA Shield (Zymo Research), and total RNA was extracted with the Quick RNA Micro kit with on-column DNase digestion (Zymo Research). Bulk RNASeq analysis was conducted as follows. Libraries with unique barcodes were prepared with the Illumina Stranded Total RNA Prep, ligated with Ribo-Zero Plus (96 Samples) kit (Cat: 20040529; Illumina), pooled at equal molar ratios, and sequenced on an Illumina NovaSeq 6000 SP sequencer to generate 150 bp paired-end reads in accordance with the manufacturer’s instructions, with a target depth of 30 million reads per sample. The FASTQ files generated were first inspected with fastqc to check sequencing quality. Sequences were aligned with the GENCODE GRCh37.p13 human reference genome with STAR aligner v2.6, and alignment quality was evaluated with RSeQC. We used Samtools to generate index files for all BAM alignments. Exon usage was determined from BAM alignment files with custom R scripts. BAM alignment files were imported into the Integrative Genomics Viewer (IGV) to analyze the splice junction reads within the *CD274* gene. Gene-level features were quantified with featureCounts v1.6.0 based on GENCODE GRCh37.p13 gene annotation. The number of transcripts per million (TPM) was determined to quantify *CD274* expression. For exon-by-exon expression analysis, the number of reads mapping to each *CD274* exon was determined, and the TPM for *CD274* was divided by the number of reads mapping to each *CD274* exon. For PCA, read-count data were normalized by variance-stabilizing transformation implemented in the DESeq2 package. DE analysis was performed with DESeq2 (Love et al., 2014). GSEA was conducted with the fgsea package by projecting genes ranked by fold-change with effect-size shrinkage (Zhu et al., 2019) onto the genesets retrieved from the MSigDB database (https://www.gsea-msigdb.org/gsea/msigdb/). Transcription factor activity inference analysis was performed with the decoupleR package and the CollecTRI gene regulatory network database (Badia-i-Mompel et al., 2022; Müller-Dott et al., 2023, *Preprint*). Raw RNASeq data were deposited in the Sequence Read Archive (SRA) under BioProject accession no. PRJNA1084900.

### Analysis of CD274 mRNA by RT-PCR and RT-qPCR

Total RNA from PBMCs for RNASeq was used to synthesize cDNA with the SuperScript IV Reverse Transcriptase (18090010; Invitrogen) and oligo-dT_16_ (N8080128; Invitrogen; for RT-PCR) or random hexamers (N8080127; Invitrogen; for RT-qPCR). Nested RT-PCR was performed with a single forward primer (5′-GCT​TCT​GTC​CGC​CTG​CA-3′) and two reverse primers (5′-AGC​CCC​GAT​GAA​CCC​CTA-3′ and 5′-TCT​TGT​CAC​GCT​CAG​CCC-3′). For RT-qPCR, two TaqMan probes targeting different exon junctions of the *CD274* mRNA (Hs01125296_m1 and Hs01125301_m1 for the exon 1–2 and 6–7 junctions, respectively) were used. *GUSB* was used as an endogenous control (4310888E; Applied Biosystems). Gene expression was quantified by the ΔΔCT method, as previously described (Ogishi et al., 2021).

### Bulk RNASeq analysis of whole-blood leukocytes

Whole-blood samples from the PD-L1-deficient siblings, their heterozygous mother, and healthy adults and age-matched controls were stabilized in Tempus Blood RNA tubes (Thermo Fisher Scientific) as previously described (Linsley et al., 2019). RNA was extracted with the MagMAX for Stabilized Blood Tubes RNA Isolation Kit and was then subjected to globin reduction with the GLOBINclear-Human Kit. Full-length cDNA was generated from total RNA (0.5 ng) and amplified with the SMART-Seq v4 Ultra Low Input RNA Kit for Sequencing (Takara Bio). Sequencing libraries were constructed with the NexteraXT DNA Library preparation Kit (Illumina). Libraries were pooled and quantified with a Qubit Fluorometer (Life Technologies). Pooled libraries were sequenced on a NextSeq 2000 sequencer (Illumina) with 59-base paired-end reads on a NextSeq P2 flow cell (Illumina), with a target depth of 5 million reads per sample. Base calls were processed to generate FASTQs on BaseSpace (Illumina), and low-confidence base calls were trimmed from read ends. FASTQs were aligned with the GRCh38 human reference genome with STAR aligner v.2.4.2a. Exon usage was determined from BAM alignment files with custom R scripts. For analysis of the TCR repertoire, the FASTQ files were analyzed with MiXCR (Bolotin et al., 2015) to reconstruct complementarity-determining region 3 (CDR3) clonotypes for the TCR α-chain (TRA) and β-chain (TRB). The TRB repertoire data derived from whole-blood genomic DNA samples from the PD-1-deficient child and his healthy brother and from three healthy donors (Adaptive Biotechnologies) from a previous study (Ogishi et al., 2021) were also reanalyzed. CDR3 length and physicochemical properties were determined from the amino-acid sequence with the alakazam package (Gupta et al., 2015). Rarefaction analysis was performed with the iNEXT package (Hsieh et al., 2016) based on clonotype count data. Metrics for the distribution of clonotype size (Gini index, mean log deviation, and the Wolfson index of bipolarization) were calculated with the dineq package. Raw RNASeq data are deposited in the SRA under BioProject accession no. PRJNA1084900.

### Immunophenotyping analysis

Fresh whole-blood leukocytes were analyzed by flow cytometry to identify and quantify the major leukocyte populations. Briefly, 200 μl EDTA-stabilized venous blood was stained with a panel of fluorochrome-conjugated antibodies (anti-CD45-PerCP [Clone: HI30; BioLegend], anti-CD3-APC/Cy7 [Clone: OKT3; BioLegend], anti-CD4-BUV395 [Clone: SK3; BD Biosciences], anti-CD8-BUV737 [Clone: SK1; BD Biosciences], anti-CD19-PE [Clone: HIB19; BioLegend], anti-CD14-Alexa Fluor 488 [Clone: HCD14; BioLegend], anti-CD16-PE/Cy7 [Clone: 3G8; BioLegend], anti-CD15-BV605 [Clone: W6D3; BioLegend], and anti-CD56-APC [Clone: HCD56; BioLegend]) for 15 min at room temperature in BD Truecount tubes. Red blood cells were lysed (BD FACS Lysing solution), and leukocytes were analyzed immediately on a BD Fortessa flow cytometer with BD FACSDiva software. Absolute cell numbers were calculated according to the manufacturer’s instructions (Truecount Tubes, BD Biosciences).

For the characterization of both rare and abundant leukocyte populations in the blood, cryopreserved PBMCs (4–8 × 10^5^ cells per individual) from the PD-L1-deficient siblings, their heterozygous mother, a healthy adult, and age-matched controls, and the previously described PD-1-deficient child (Ogishi et al., 2021) were analyzed by spectral flow cytometry as previously described (Ogishi et al., 2023).

### scRNASeq analysis

Cryopreserved PBMCs from the PD-L1-deficient siblings, their heterozygous mother, one age-matched healthy control, and two adult healthy controls were subjected to scRNASeq analysis as previously described (Ogishi et al., 2022). In addition, PBMCs from two APDS patients and one STAT3 GOF patient were analyzed as disease controls. Previously obtained data for healthy and diseased controls, including the PD-1-deficient child and his healthy brother (Ogishi et al., 2021) and one RNaseL-deficient MIS-C patient (Lee et al., 2023), were also integrated into the analysis. Gene regulatory network (GRN) analysis was conducted with the SCENIC pipeline (Aibar et al., 2017). Predicted single-cell regulon activities were aggregated by individual and leukocyte subsets. Wilcoxon’s rank-sum test was used to compare aggregated regulon activities between age-matched controls and individuals with PD-1 and PD-L1 deficiencies. The regulons with the lowest P values (ties allowed) were selected for visualization. Raw scRNASeq data were deposited in the SRA under BioProject accession no. PRJNA1084900. Preprocessed scRNASeq data and analysis scripts were deposited in the Mendeley Data Repository (https://doi.org/10.17632/4fwbcswj8d.1). Integrated historical data are available under BioProject accession nos. PRJNA818002, PRJNA723618, PRJNA898284, PRJNA936917, and PRJNA845112.

### Analysis of cytokine production by PD-L1- and PD-1-deficient T-blasts

T-blasts (2 × 10^5^ cells per well) from the PD-L1-deficient siblings, their heterozygous mother, one age-matched control, several adult controls, and the PD-1-deficient child and his healthy brother were analyzed. The cells were either left non-stimulated or were stimulated with Dynabeads Human T-Activator CD3/CD28 (Invitrogen, T:Bead = 1:1), ImmunoCult Human CD3/CD28/CD2 T Cell Activator (1:100; STEMCELL Technologies), or Cell Stimulation Cocktail (1:1,000; eBioscience). For the PD-1:PD-L1 blockade assay, the following mAbs were added at a final concentration of 5 μg/ml: anti-PD1 mouse IgG1 (Cat: A01829, Clone: PD1.D3; GenScript), mouse IgG1 isotype control (Cat: MAB002, Clone: #11711; R&D), anti-PD-L1 human IgG1 (N298A) (Cat: hpdl1-mab12; InvivoGen, equivalent to atezolizumab), and human IgG1 isotype control (N298A) (Cat: bgal-mab12; InvivoGen). We quantified cytokine secretion with the LEGENDplex kit (BioLegend, Human CD8/NK Panel or Human T Helper Cytokine Panels Version 2). For flow cytometry analysis, monensin and brefeldin A (1:1,000 each; Cytek) were added. The cells were collected, stained with Zombie NIR Fixable Viability Kit (1:2,000; BioLegend), surface-stained with lineage markers, and fixed and permeabilized with the Foxp3/Transcription Factor Staining Buffer Set (Cytek). Cells were then stained by incubation overnight at 4°C in the dark with the following reagents in permeabilization buffer: FcR blocking reagent (1:50; Miltenyi Biotec), anti-CD3-FITC (Clone: UCHT1, 1:100; Cytek), anti-IL-2-BV510 (Clone: MQ1-17H12, 1:100; Cytek), anti-IFN-γ-PE-Dazzle 594 (Clone: 4S.B3, 1:250; BioLegend), and anti-TNF-BV711 (Clone: MAb11, 1:500; BioLegend) mAbs. The cells were washed with FACS buffer and acquired with an Attune NxT Flow Cytometer with the CytKick MAX Autosampler (Invitrogen). Data were analyzed using FlowJo and R software.

### Statistical analysis

All statistical analyses were performed in R v. 4 (http://www.R-project.org/) (R Core Team, 2021). The statistical significance of quantitative differences between groups was assessed in two-tailed unpaired Wilcoxon’s rank-sum tests unless otherwise stated. FDR adjustment was performed using the Benjamini and Hochberg method (Benjamini and Hochberg, 1995). P values below 0.05 were considered statistically significant.

### Online supplemental material

The online supplementary information describes the ancestry of PD-L1-deficient P1 and P2 and their parents (Fig. S1), an in-depth analysis of *CD274* mRNA in the leukocytes of P1 and P2 (Fig. S2), an immunophenotyping analysis of leukocytes in P1 and P2 (Fig. S3), a comparative analysis of PD-1- and PD-L1-deficient leukocyte subsets through scRNASeq (Fig. S4), and an analysis of cytokine production by PD-1- and PD-L1-deficient T lymphocytes stimulated in vitro (Fig. S5). Table S1 describes the quality control metrics of whole-genome sequencing for the affected siblings and their parents. Table S2 lists homozygous non-synonymous variants common to the two affected siblings. Table S3 lists all genes known to underlie IEI. Table S4 describes heterozygous variants in genes known to underlie IEI and common to the two affected siblings. Table S5 describes de novo variants in P1 or P2. Table S6 describes genes within the 7q11.23 duplicated region in P1. Table S7 lists all differentially expressed genes in PD-1- and PD-L1-deficient patients, and patients with monogenic etiologies of autoinflammatory and autoimmune diseases compared with age-matched healthy donors.

## Supplementary Material

Table S1 shows whole-genome sequencing metrics.

Table S2 shows homozygous non-synonymous variants common to the two affected siblings.

Table S3 shows genes known to underlie IEI.

Table S4 shows heterozygous variants in genes known to underlie IEI and common to the two affected siblings.

Table S5 shows de novo variants in P1 or P2.

Table S6 shows genes within the 7q11.23 duplicated region in P1.

Table S7 shows differentially expressed genes in PD-1- and PD-L1-deficient patients, and patients with monogenic etiologies of autoinflammatory and autoimmune diseases, compared with age-matched healthy donors.

SourceData F2 is the source file for Fig. 2.

SourceData F3 is the source file for Fig. 3.

SourceData F4 is the source file for Fig. 4.

## Data Availability

Raw bulk RNASeq and scRNASeq datasets generated in this study are deposited in the SRA under BioProject accession no. PRJNA1084900. Preprocessed scRNASeq data and analysis scripts are deposited in the Mendeley Data repository (https://doi.org/10.17632/4fwbcswj8d.1). All other raw and processed data are available from the corresponding authors upon request.

## Data availability

Raw bulk RNASeq and scRNASeq datasets generated in this study are deposited in the SRA under BioProject accession no. PRJNA1084900. Preprocessed scRNASeq data and analysis scripts are deposited in the Mendeley Data repository (https://doi.org/10.17632/4fwbcswj8d.1). All other raw and processed data are available from the corresponding authors upon request.
